# Isolation and Biological Evaluation of Human Tyrosinase Inhibitors from the Fruit of *Xanthium strumarium* L.

**DOI:** 10.3390/molecules30183689

**Published:** 2025-09-10

**Authors:** Gengxuan Shi, Yaoying Lu, Yougang Zhang, Ke Zheng, Jean Giacomotto, Kathryn F. Tonissen, Yunjiang Feng

**Affiliations:** 1 Institute for Biomedicine and Glycomics, Griffith University, Nathan, QLD 4111, Australia; maxxie.shi@griffithuni.edu.au (G.S.); y.lu@griffith.edu.au (Y.L.); yougang.zhang@griffithuni.edu.au (Y.Z.); j.giacomotto@griffith.edu.au (J.G.); k.tonissen@griffith.edu.au (K.F.T.); 2 School of Environment and Science, Griffith University, Nathan, QLD 4111, Australia; ke.zheng@griffithuni.edu.au; 3 Queensland Brain Institute, The University of Queensland, Brisbane, QLD 4067, Australia

**Keywords:** *Xanthium strumarium* L., human tyrosinase inhibitor, depigmenting agent, 4-hydroxybenzoic acid, melanin reduction, tyrosinase assay

## Abstract

Tyrosinase catalyzes the rate-limiting steps of melanin production, posing as an important target for treating skin pigmentation. This study investigates bioactive human tyrosinase inhibitors from *Xanthium strumarium* L. using a combined strategy of cell lysate, cell-based, and zebrafish assays. In this study, the methanol extract of *Xanthium strumarium* L. was identified as a potent inhibitor against tyrosinase in a cell lysate assay utilizing human MM418C1 melanoma cells. Subsequent phytochemical analysis resulted in the isolation of 11 natural products, including 4-hydroxybenzoic acid (4HB), three nucleotides, four caffeoylquinic acids and three alkaloids. Biological activity evaluation of isolated compounds suggested that 4HB was a potent inhibitor against tyrosinase with an IC_50_ value of 59.5 μg/mL. Further evaluations revealed that 4HB significantly reduced the melanin content by 40% at the concentration of 500 mg/mL in human MM418C1 melanoma cells. 4HB activity was finally confirmed in vivo, by the demonstration of 40% reduction in melanin production in live zebrafish at the concentration of 15.63 μg/mL.

## 1. Introduction

Human (*Homo sapiens*) tyrosinase (*hs*TYR) is one of the key enzymes involved in melanogenesis and represents a major therapeutic target for depigmenting agents [1]. Structurally, *hs*TYR consists of a single-chain glycoprotein with a binuclear copper centre in its active site, crucial for its catalytic activity [2,3]. It initiates and regulates melanogenesis through two critical reactions: the hydroxylation of tyrosine to L-3,4-dihydroxyphenylalanine (L-DOPA) and the subsequent oxidation of L-DOPA to dopaquinone, which undergoes further polymerization to form melanin [4].

Several tyrosinase inhibitors, such as hydroquinone, arbutin, kojic acid, and azelaic acid, are used in the cosmetic and pharmaceutical industries for hyperpigmentation [5,6]. Hydroquinone is a highly potent inhibitor that directly binds to tyrosinase. However, it causes skin irritation, allergic reactions and long-term toxicity [7]. Arbutin, a glycosylated derivative of hydroquinone, offers a safer alternative with milder side effects but requires higher concentrations to achieve comparable efficacy [8]. Kojic acid, a natural compound derived from fungal fermentation, inhibits tyrosinase by chelating copper ions and is valued for its natural origin, although it can cause skin sensitivity and instability in formulations [9]. Azelaic acid, a non-competitive tyrosinase inhibitor, showed only limited potency [10] with side effects such as skin burning, stinging and redness [11]. Consequently, there is a critical need for the development of safer and more effective *hs*TYR inhibitors.

In our continuing effort to discover *hs*TYR inhibitors, a cell lysate assay was developed [12] and used to screen natural product extracts for potential *hs*TYR inhibitors. Our results showed that the methanol extract of *Xanthium strumarium* L., a traditional Chinese medicine (TCM), inhibited *hs*TYR activity by 52% at 1 mg/mL concentration.

*Xanthium strumarium* L. (*X. strumarium*), commonly known as cocklebur, a plant from the *Asteraceae* family, has been extensively used in TCM for its therapeutic effects, particularly in treating skin and respiratory conditions [13]. It is widely distributed across the northeast and northwest of China [14]. In TCM, *X. strumarium* is commonly employed to dispel wind-heat and dampness, which are believed to contribute to melasma and freckles, and to improve overall skin tone and clarity [15].

Over 170 distinct compounds have been identified from *X. strumarium*, including sesquiterpene lactones [16,17,18,19], flavonoids [20,21], steroids [22,23,24], phenylpropenoids [19,20,25,26,27] and glycosides [28,29]. Sesquiterpene lactones constitute a prominent and abundant class of natural compounds, with approximately 50 distinct compounds identified [30]. Notable sesquiterpene lactones such as xanthinin, xanthumin, iso-xanthinin, and xantholactone, have shown significant anti-inflammatory and anticancer effects [15]. Chlorogenic acid and caffeic acid are the primary phenolic acids identified from *X. strumarium*. They are known for their potent antioxidant properties, resulting in scavenging free radicals and reducing oxidative stress [31]. These compounds also contribute to the plant’s ability to modulate inflammatory responses and protect cells from oxidative damage [32,33]. The plant also contains a variety of flavonoids, including derivatives of quercetin, which enhance its antioxidant and anti-inflammatory properties [34,35]. However, little research has been performed investigating its skin depigmentation properties. Although the anti-melanogenic activity of the crude methanolic extract of *X. strumarium* fruit has been previously investigated using the mouse Mel-Ab melanocyte cell line [13], the specific bioactive constituents responsible for this activity remain unidentified. In this study, a phytochemical analysis of *X. strumarium* fruit extract was conducted to identify the components that contribute to its *hs*TYR inhibitory activity. Cell lysate and cell-based assays using human MM418C1 melanoma cells were employed to evaluate the anti-*hs*TYR activity. Additionally, bioactive compounds isolated from *X. strumarium* were tested in vivo using the zebrafish animal model to confirm their depigmentation properties.

## 2. Results

### 2.1. Isolation of Natural Products from the Methanol Extract of X. strumarium Fruit

**Figure 1:**
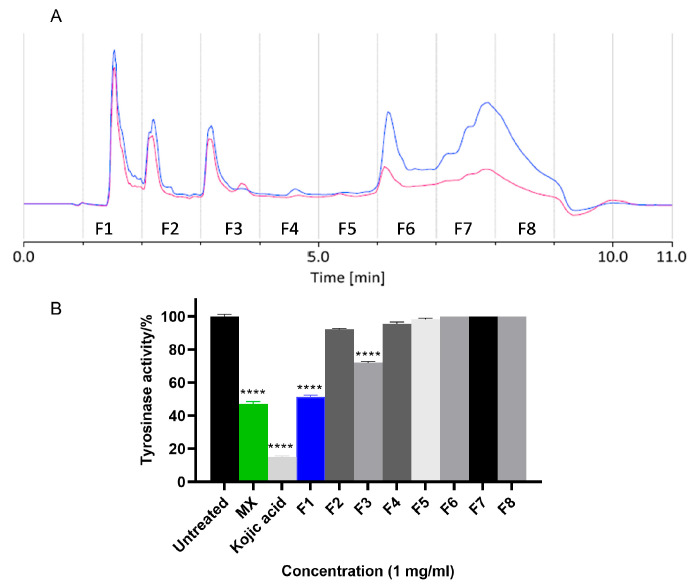
(**A**) HPLC chromatogram of methanol extract (MX). Eight fractions were collected, 1 min per fraction. The blue trace was the chromatogram at 254 nm while the red one was at 320 nm. (**B**) TYR inhibitory activity of MXs and fractions in cell lysate *hs*TYR assays. Cell lysate containing 3 × 10^6^ human MM418C1 melanoma cells were incubated with MX/the fractions (1 mg/mL) for 1 h. The tyrosinase activity was presented as a percentage of untreated group. Kojic acid (1 mg/mL) was used as positive control. Mean ± SEM (*n* = 3) of three independent experiments, each performed in triplicate were shown. One-way ANOVA was employed followed by Dunnett’s test. **** *p* < 0.0001.

The fruit of *X. strumarium* was sequentially extracted with hexane, DCM and methanol. The extracts were then tested for their inhibitory activity against *hs*TYR via cell lysate assay [12]. Methanol extract (MX) significantly reduced *hs*TYR activity (Figure 1B) by approximately 50% at 1 mg/mL. To facilitate the isolation of bioactive compounds, MX was further fractionated using Onyx Monolithic C18 column to obtain 8 fractions (F1 to F8, Figure 1A). The activity evaluation (Figure 1B) showed that F1 and F3 significantly inhibited *hs*TYR activity by 49% and 28% at 1 mg/mL, respectively.

**Figure 2:**
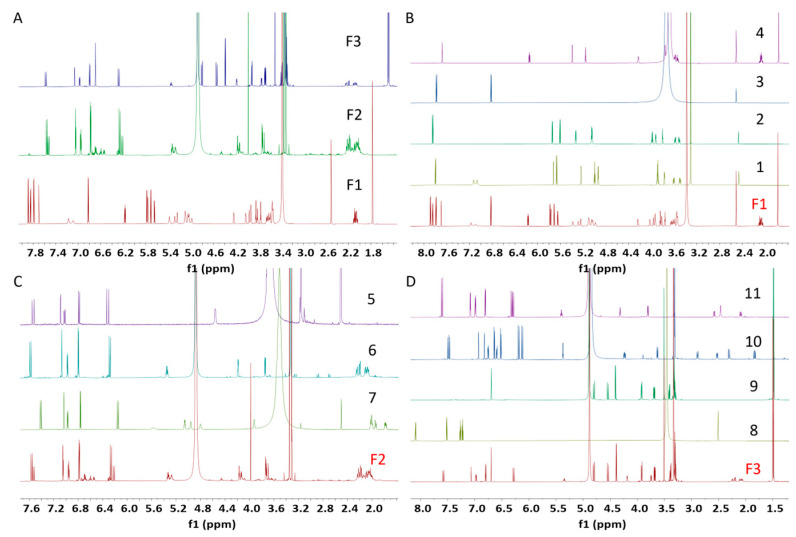
^1^H NMR spectra (800 MHz, DMSO-d_6_ or CD_3_OD-d_4_) for (**A**) F1, F2 and F3; (**B**) F1, 4-hydroxybenzoic acid (**1**), uridine (**2**), thymidine (**3**), cytidine (**4**); (**C**) F2, caffeoyl choline (**5**), 3-caffeoylquinic acid (**6**), and 5-caffeoylquinic acid (**7**); (**D**) F3, indole-3-carboxaldehyde (**8**), xanthiside (**9**), 1, 5-dicaffeoylquinic acid (**10**) and 1, 3-dicaffeoylquinic acid (**11**).

**Figure 3:**
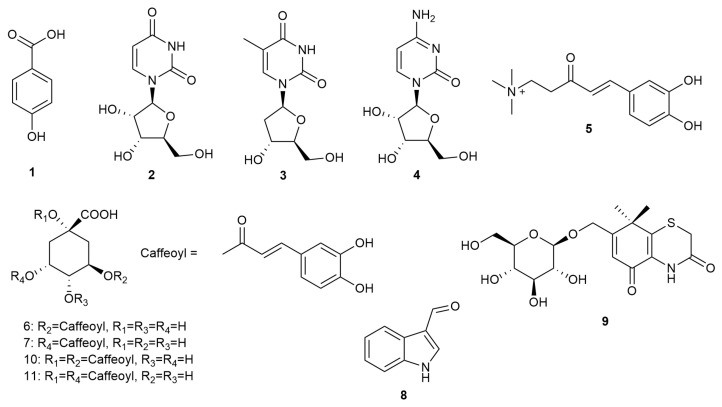
Chemical structures of compounds **1**–**11** isolated from the methanol extract of *Xanthium strumarium* L.

^1^H NMR fingerprinting, a useful strategy to target molecules of interest, was employed throughout the natural product isolation. Specifically, ^1^H NMR data of the active fractions F1 and F3 implied that different types of compounds were present in these fractions (Figure 2A). For example, fraction F1 displayed signals for sugar moieties at δ_H_ 3.5–4.2 and olefinic protons at δ_H_ 5.0–5.7 (Figure 2A), indicating the presence of nucleotides, a well-known class of natural products from *X. strumarium*. In addition, several aromatic protons were also observed at δ_H_ 6.2–8.0 in the active fraction. Repeated HPLC purification of fraction F1 led to the isolation of 4-hydroxybenzoic acid (**1**, 11.4 mg, 0.023% of dry wt.), and three nucleotides, including uridine (**2**, 9.8 mg, 0.020% of dry wt.), thymidine (**3**, 7.1 mg, 0.014% of dry wt.), and cytidine (**4**, 12.6 mg, 0.025% of dry wt.) (Figure 3). The ^1^H NMR signals of the 4 compounds matched well with those in F1 (Figure 2B), illustrating that the major compounds have been isolated from the active fraction F1.

The ^1^NMR spectrum of F2 indicated the presence of caffeoyl functional group with characteristic signals at δ_H_ 6.3–7.7 and series of CH_2_ groups of quinic acid ring system at δ_H_ 1.8–2.4 (Figure 2C). Further purification resulted in the isolation of caffeoyl choline (**5**, 15.7 mg, 0.031% of dry wt.), 3-caffeoylquinic acid (**6**, 5.1 mg, 0.011% of dry wt.), and 5-caffeoylquinic acid (**7**, 12.1 mg, 0.024% of dry wt.) (Figure 3).

^1^H NMR spectrum of active fraction F3 displayed fingerprint signals for aromatic protons at δ_H_ 6.2–7.8 and set of signals at δ_H_ 3.9–5.0. The purification was guided by ^1^H NMR spectral data, yielding indole-3-carboxaldehyde (**8**, 0.2 mg, 0.0004% of dry wt.), xanthiside (**9**, 14.1 mg, 0.028% of dry wt.), 1, 5-dicaffeoylquinic acid (**10**, 0.7 mg, 0.0014% of dry wt.) and 1, 3-dicaffeoylquinic acid (**11**, 0.5 mg, 0.001% of dry wt.), as well as the re-isolation of compound **7** (Figure 2D) (Figure 3).

Compounds **1**–**11** (Figure 3) have been previously isolated from the stem and leaves of *X. strumarium* [36,37], and their 1D and 2D NMR data and mass spectroscopic data obtained in this study were consistent with the data reported in the literature [28,38,39,40,41,42].

### 2.2. Biological Activity of MX

#### 2.2.1. Inhibitory Activity of MX Against *hs*TYR

**Figure 4:**
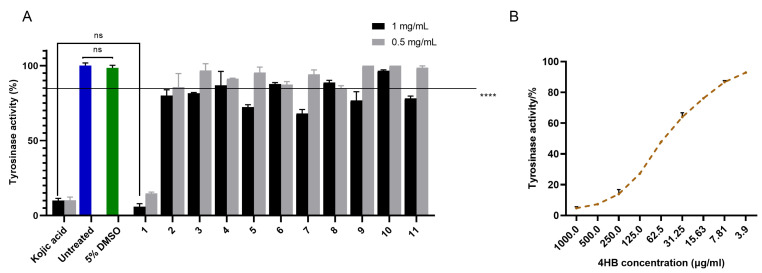
(**A**) Anti-*hs*TYR activity of compounds **1**–**11** using cell lysate assays. Cell lysates prepared from 3 × 10^6^ MM148C1 human melanoma cells were incubated with 1 or 0.5 mg/mL of compounds **1**–**11** in 5% DMSO for 1 h. Kojic acid was used as a positive control. TYR activity is presented as a percentage of 5% DMSO group (negative control). The cutoff line represents the threshold value for **** *p* < 0.0001 (threshold: 85.0198%), ns: not statistically significant. (**B**) Dose–response curve of 4HB against *hs*TYR. Cell lysate prepared from 3 × 10^6^ MM148C1 human melanoma cells were incubated with 1000.0, 500.0, 250.0, 125.0, 62.5, 31.3, 15.6, 7.8 and 3.9 μg/mL 4HB solution for 1 h. Mean ± SEM (*n* = 3) of three independent experiments, each performed in triplicate are shown. Two-way ANOVA was employed followed by Šídák’s multiple comparisons test.

The *hs*TYR inhibitory activity of the 11 isolated compounds was first investigated via the cell lysate assay using the human MM418C1 melanoma cell line (Figure 4). At 1 mg/mL, compounds **1** (5.85%), **2** (80.12%), **5** (72.41%), **7** (68.08%), **9** (76.86%) and **11** (78.10%) significantly decreased *hs*TYR activity, while at 0.5 mg/mL, only compound **1** (14.73%) was active with 85.27% reduction in hsTYR activity. The activity was comparable to kojic acid, the positive control. IC_50_ was determined to be 57.14 ± 5.25 μg/mL (equivalent to 413.7 ± 38.0 μM) for compound **1** (Figure 4B), in comparison to 67.07 ± 12.22 μg/mL (equivalent to 472.0 ± 86.0 μM) for kojic acid.

#### 2.2.2. Cell Proliferation Test for MX and 4-Hydroxybenzoic Acid (4HB)

**Figure 5:**
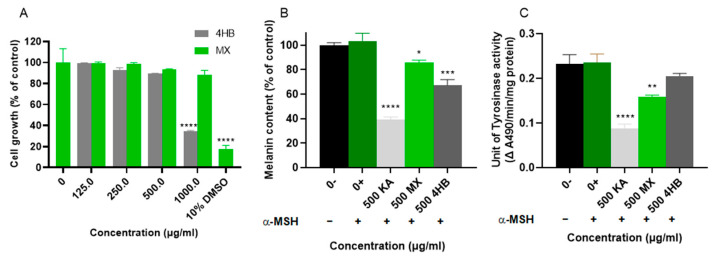
Cell-based *hs*TYR assay for 4HB. (**A**) Cell viability test (resazurin assay). Human MM418C1 melanoma cells were treated with 1000.0, 500.0, 250.0 and 125.0 μg/mL 4HB/MX. The growth of the cells is expressed as a percentage of the untreated group. Human MM418C1 melanoma cells were treated with 500 μg/mL 4HB (500 4HB) or MX (500 MX) and 0.1 μM α-MSH for 5 days to assess its effect on (**B**) melanin content and (**C**) cellular TYR activity. Cells with growth media (0−) were used for comparison with the α-MSH treated cells (0+). 500 μg/mL kojic acid (500 KA) was utilized as a positive control in both assays. The melanin content was determined using the percentage of the untreated group. The unit of TYR activity was determined by following the change in Aλ490/min/mg protein. Mean ± SEM (*n* = 3) of three independent experiments, each performed in triplicate, are shown. One-way ANOVA was employed followed by Dunnett’s test. **** *p* < 0.0001, *** *p* < 0.001, ** *p* < 0.01, * *p* < 0.05.

The tyrosinase inhibitory effect of compound **1**, 4-hydroxybenzoic acid (4HB), on pigmented human MM418C1 melanoma cells was also investigated. Additionally, the bioactivity of MX was evaluated to assess its inhibitory activity against *hs*TYR as a whole extract. First, the toxicity of 4HB and MX on MM418C1 cells was determined via resazurin assays. Cells were treated with 1000.0, 500.0, 250.0, 125.0 and 62.5 μg/mL 4HB or MX. The cell growth was then assessed after 5 days. 10% DMSO treated cells were utilized as a positive control for cytotoxicity. Figure 5A showed that only 1000 μg/mL 4HB resulted in 60% cell death. When cells were treated with 500 μg/mL and lower concentrations of 4HB, no significant reduction in cell proliferation was observed. However, no toxicity data was observed for MX at concentrations less than 1000 μg/mL. Therefore, 500 μg/mL was chosen for further experiments.

#### 2.2.3. Cell-Based *hs*TYR Assay and Melanin Assay for MX and 4HB

The anti-melanogenic activity of 4HB and MX was also investigated in human MM418C1 melanoma cells. Melanin content assay and cellular TYR assay were performed on human MM418C1 melanoma cells. Alpha-melanocyte-stimulating hormone (α-MSH) is a known stimulator of melanogenesis [43] and has been utilized in this human cell model. The primary rationale for using α-MSH is to induce melanogenesis and enhance melanin production in the cells, as melanoma cells exhibit reduced pigmentation with continued subculturing [44]. Cells were treated with 500 μg/mL of 4HB, MX, or kojic acid for 5 days, and their effects on melanin content and cellular TYR activity were analyzed. Treatment with 4HB resulted in a significant reduction in melanin content (Figure 5B), with approximately 33% decrease observed; however, there was no significant reduction in cellular TYR activity (Figure 5C). MX treatment resulted in a much smaller effect, reducing melanin content by less than 15% while decreasing TYR activity by approximately 35%. In contrast, kojic acid reduced both melanin content and TYR activity by approximately 60%, consistent with the literature [45,46].

### 2.3. Zebrafish Pigmentation Assays for MX and 4HB

**Figure 6:**
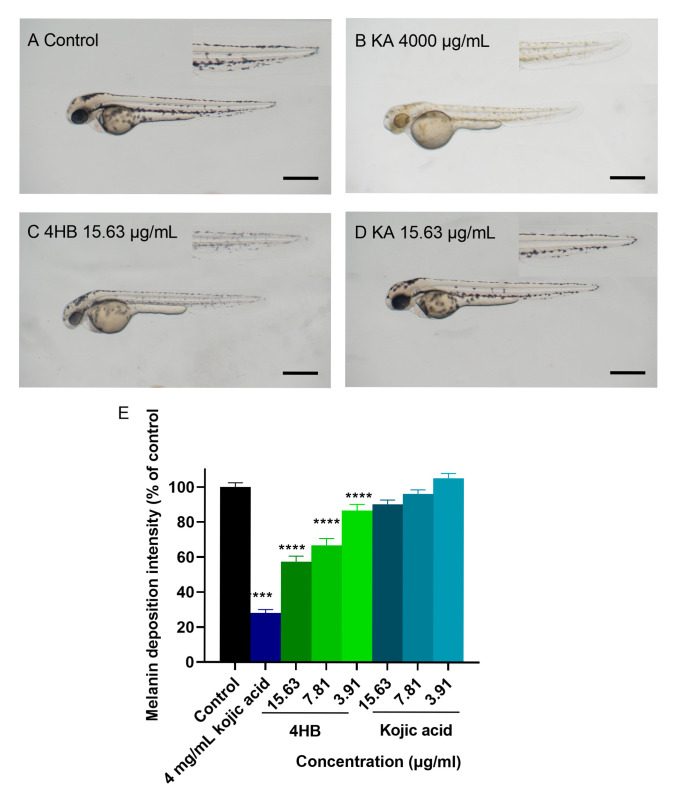
Zebrafish pigmentation assays for MX and 4HB. Synchronized embryos were treated with 4HB and kojic acid at a concentration of 15.63, 7.81 and 3.91 μg/mL. Kojic acid (KA) at 4 mg/mL was used as a positive control and standard E3 medium was used as a negative control. Tested compounds were dissolved in embryo E3 medium. Zebrafish pigmentations were assessed using a stereomicroscope coupled with digital camera for in silico processing/quantification of the pigmented areas. (**A**–**D**) lateral view and magnified caudal fin view of embryos at 48 h post-fertilization (hpf). (**E**) statistical analysis of pigmentation displayed as percentage to untreated control group in zebrafish (*n* = 9). Image and contrast-based analysis were conducted in Fiji ImageJ 2.9.0 software; One-way ANOVA was employed followed by Dunnett’s test. **** *p* < 0.0001 versus control. Scale bar = 100 μm.

The anti-melanogenic activity of 4HB and MX was further investigated in vivo using the zebrafish animal model. Zebrafish develops significant pigmentation in less than 48 h of fertilization, which enables rapid investigation of potential depigmentation agents. To control our readouts, we used kojic acid at 4 mg/mL as positive control and standard E3 medium as negative control. Toxicity evaluation (Supplementary Figure S1) suggested that 4HB was toxic to zebrafish at concentrations higher than 15.63 μg/mL (less than 80% survival), however it showed minimal toxicity at 15.63, 7.81 and 3.91 μg/mL, in contrast, kojic acid had little toxicity at concentrations up to 4 mg/mL. For comparison, the depigmentation experiment on zebrafish was carried out by using doses ranging from 3.91 to 15.63 μg/mL of 4HB and kojic acid (Figure 6). The results suggested that at lower concentrations ranging from 15.83 to 7.81 μg/mL, kojic acid had no significant effect on pigmentation in zebrafish. In contrast, 4HB reduced zebrafish embryos pigmentation by 40%, 35% and 20% at the concentrations of 15.83, 7.81 and 3.91 μg/mL, respectively (Figure 6E). However, it is noteworthy that kojic acid at 4000 μg/mL significantly reduced pigmentation by almost 75% (Figure 6B,E).

## 3. Discussion

In this research, 4HB was identified as the most active tyrosinase inhibitor from the fruit of *X. strumarium*. Interestingly, when tested in vivo using the zebrafish animal model, 4HB demonstrated a greater depigmenting activity than the widely used kojic acid, suggesting an attractive potential for clinical application (Figure 6E). However, despite its significant reduction in cellular melanin production, 4HB exhibited little inhibitory activity against cellular *hs*TYR, suggesting that the mechanism underlying 4HB’s inhibitory effect on melanin production may not directly involve *hs*TYR. It is worth noting that 4HB showed significant toxicity in zebrafish at concentrations greater than 15.6 μg/mL. It may be due to its acidity with a dissociation constant (pKa) of 4.58 [47], in comparison to 7.90 for kojic acid [48]. This lower pKa suggests that 4HB can more readily donate protons in aqueous environments, thereby potentially causing a more acidic local environment in zebrafish assay. [49]. There is limited direct literature on the depigmenting effects of 4HB. However, it is a metabolite of parabens and has been studied for its role in phenolic content and related antioxidant properties, which could influence skin health and pigmentation [50,51]. It is also known for its low toxicity and estrogenic activity in certain contexts [52], but its direct efficacy in melanogenesis inhibition or depigmentation has not been prominently highlighted in recent studies. Therefore, this is the first report evaluating the anti-*hs*TYR activity of 4HB using both human melanoma cells and an in vivo zebrafish model. While 4HB exhibited lower potency compared to oxyresveratrol—one of the well-established natural *hs*TYR inhibitors identified using a recombinant *hs*TYR assay [53]—our findings demonstrate that 4HB retains measurable activity in a whole-organism system. Importantly, the observed depigmenting effect of 4HB in the zebrafish model supports its transdermal permeability and whole-organism efficacy, thereby advancing current knowledge on its potential as a lead compound for anti-hyperpigmentation therapies.

Apart from 4HB, other compounds isolated from the methanol extract of *X. strumarium* such as thymidine (**3**), caffeoyl choline (**5**), 5-caffeoylquinic acid (**7**) and 1, 3-dicaffeoylquinic acid (**11**) also showed significant inhibitory activity against *hs*TYR, contributing to the overall depigmenting effects of *X. strumarium*. The results suggests that the whole methanol extract of *X. strumarium* fruit can be a potential treatment for skin pigmentation. This aligns with the traditional use of *X. strumarium* in TCM, where the whole alcoholic extract is typically utilized [54]. In many TCM practices, combinations of several medicinal herbs are often prescribed to enhance therapeutic effects [55]. Therefore, using the extract of *X. strumarium* could provide a more holistic approach, similar to its traditional application, potentially leading to more effective formulations for skin depigmentation.

Our results have demonstrated that the combination of in vitro human melanoma cell lysate- and cell-based assays, combined with the in vivo zebrafish assay, offers an effective approach for identifying and assessing *hs*TYR inhibitors. This integrated strategy enhances the discovery process by providing a comprehensive evaluation of both enzyme inhibition and biological efficacy. Notably, one compound with effective inhibitory activity against melanin production, 4HB, was discovered from the fruit of *X. strumarium*. However, the precise inhibitory mechanism of 4HB remains to be thoroughly elucidated, necessitating more in-depth investigation to fully understand its mode of action.

## 4. Materials and Methods

### 4.1. General Experimental Procedures

The fruit of *X. strumarium.* was purchased from Hong Ren Tang Herbal, Sunnybank, Australia. L-glutamine, penicillin/streptomycin, trypsin, RPMI 1640 medium (powder) were purchased from Life Technologies, Carlsbad, CA, USA. Bovine serum albumin (BSA), L-dopa, sodium phosphate, kojic acid, arbutin, Triton X-100 and silica gel were obtained from Sigma Chemicals, Castle Hill, NSW, Australia. Fetal bovine serum (FBS) was purchased from Bovogen Biologicals, Keilor East, VIC, Australia. Trypan blue 0.4% solution was purchased from MP biomedicals, Seven Hills, VIC, Australia. HPLC was carried out on a Thermo Scientific (Waltham, MA, USA) UtiMate 3000 System with gradient acidic MeOH/H_2_O elution (0.1% *v*/*v* trifluoroacetic acid). NMR spectra were carried out on Brucker (Billerica, MA, USA) Avance HDX 800 MHz spectrometer at 25 °C. Solvent signals were referenced for CD_3_OD-*d*_4_ (δ_H_ 3.31, δ_C_ 49.0) and DMSO-*d*_6_ (δ_H_ 2.50, δ_C_ 39.50). Both 1D-(^1^H and ^13^C) and 2D-NMR (COSY, HSQC, HMBC and ROESY) spectra were analyzed through MestReNova software (Version 11.0.2-18153). LC-MS were carried out on the Thermo UtiMate 3000 System coupled with C18 column (2.6 μm, 150 × 2.1 mm) with gradient MeOH/H_2_O elution (0.1% *v*/*v* formic acid).

### 4.2. Extraction

The dry powder of fruits of *X. strumarium* (49.50 g) were ground and extracted by immersing in hexane on the shaker (2 × 300 mL, 2 h each). The residue was then extracted by dichloromethane (DCM) (2 × 300 mL, 2 h each) and methanol (2 × 300 mL, 2 h each) sequentially. Solvents were evaporated to generate hexane (2.71 g), DCM (0.98 g) and methanol (1.01 g) extract (MX), respectively. The extraction process was conducted at 25 °C using a solvent-to-solid ratio of 12.12 mL/g (for each solvent).

### 4.3. Isolation and Purification

For small-scale fractionation, the MX (19.02 mg) was first dissolved in methanol and uniformly applied onto a small piece of cotton. The cotton was dried under a constant air flow and packed into a cartridge. This cartridge was then connected to an Onyx monolithic C18 column (100 × 4.6 mm, 5 μm particle size, Phenomenex, Lane Cove, NSW, Australia) and eluted at a flow rate of 4 mL/min. Gradient elution was applied with MeOH/H_2_O (10:90) to 100% MeOH (with 0.1% trifluoroacetic acid, TFA) within 6 min, then 100% MeOH for 1 min, then to 10% MeOH within 1 min, and maintain at 10% MeOH for 3 min. 8 fractions were obtained (F1: 1–2 min, F2: 2–3 min, F3: 3–4 min, F4: 4–5 min, F5: 5–6 min, F6: 6–7 min, F7: 7–8 min, F8: 8–9 min) and were tested at 1 mg/mL.

Large scale isolation was achieved by reverse-phase HPLC using various solvent systems under the guidance of ^1^H NMR fingerprinting. Specifically, MX (42.13 mg) was dissolved in methanol and evenly applied to a small piece of cotton. After the methanol was completely evaporated, the cotton was packed into a cartridge, which was then connected to a Betasil C18 column (150 × 21.2 mm, 5 μm particle size, from Thermo, Scoresby, VIC, Australia) eluting at a flow rate of 9 mL/min with linear gradient MeOH/H_2_O (10:90) to 70% MeOH within 50 min, then to 100% MeOH within 5 min, then maintain for 5 min. Fractions collected between 1 and 25 min were combined and subjected to further purification using a Luna C18 column (250 × 10 mm, 5 μm particle size, from Phenomenex, Lane Cove, NSW, Australia) at a flow rate of 4 mL/min. The elution was conducted using a linear gradient of MeOH/H_2_O (10:90) (0.1% TFA) to 40% MeOH within 60 min, resulting in the isolation of 4-hydroxybenzoic acid (**1**, 11.4 mg, 0.023% of dry wt.), along with uridine (**2**, 9.8 mg, 0.020% of dry wt.), thymidine (**3**, 7.1 mg, 0.014% of dry wt.), and cytidine (**4**, 12.6 mg, 0.025% of dry wt.). Similarly, fractions eluted between 26 and 29 min were pooled and further purified using the same Luna C18 column under identical flow conditions. The gradient ranged from MeOH/H_2_O (20:80) (0.1% TFA) to 40% MeOH over 60 min, which led to the isolation of caffeoyl choline (**5**, 15.7 mg, 0.031% of dry wt.), 3-caffeoylquinic acid (**6**, 5.1 mg, 0.011% of dry wt.), and 5-caffeoylquinic acid (**7**, 12.1 mg, 0.024% of dry wt.). Furthermore, fractions collected between 30 and 40 min underwent additional purification on the Luna C18 column, using a linear gradient from MeOH/H_2_O (30:70) containing 0.1% TFA to 55% MeOH over 60 min at the same flow rate. This purification step facilitated the isolation of indole-3-carboxaldehyde (**8**, 0.2 mg, 0.0004% of dry wt.), xanthiside (**9**, 14.1 mg, 0.028% of dry wt.), 1, 5-dicaffeoylquinic acid (**10**, 0.7 mg, 0.0014% of dry wt.) and 1, 3-dicaffeoylquinic acid (**11**, 0.5 mg, 0.001% of dry wt.).

### 4.4. Cell Culture

The human MM418C1 melanoma cell line was used for assays. MM418C1 is a clone of the MM418 melanoma cell line that originated from the primary lesion of the cutaneous surface [56].

The human MM418C1 melanoma cell line was cultured in RPMI 1640 medium, which contained 10% (*v*/*v*) FBS, 200 mM L-glutamine, 100 U/mL penicillin, and 100 g/mL streptomycin. A T-75 flask was used to keep the cells at 37 °C, in a 5% CO_2_ incubator, and cells were subcultured every two days. The number of viable cells in the cell suspension was determined using trypan blue exclusion. To count the cells, 10 μL of cell suspension were mixed with 10 μL of 0.4% (*w*/*v*) trypan blue solution. Then, 10 μL of the mixture was applied to a hemocytometer and total viable cells counted.

### 4.5. Protein Estimation

Bovine serum albumin (BSA) was used as standard and 10 mg/mL of BSA solution was used to prepare 2 mg/mL, 1 mg/mL, 0.5 mg/mL, 0.25 mg/mL and 0.125 mg/mL BSA solutions by serial dilution. Then, 10 μL of each BSA solution was transferred to a 96-well plate in triplicate to obtain a standard curve. The cell extracts were diluted 1/20 and 10 μL of the diluted cell extracts were transferred to appropriate wells of a 96-well plate in triplicate. The DC protein assay kit (Bio-Rad, Sydney, NSW, Australia) was used to determine the protein concentration. Firstly, 20 μL of reagent S was mixed with 1 mL of reagent A. Then 25 μL of this mixture was added to each well and incubated for 1 min at room temperature. Then, 200 μL of reagent B was added to each well. The 96-well plate was mixed in the SpectraMax M3 plate reader (Bio-strategy, Melbourne, VIC, Australia) for 5 min and then left in the dark for 15 min. The absorbance was measured at 750 nm. The protein concentration of each sample was calculated using the BSA standard curve.

### 4.6. Cell Lysate Based hsTYR Assay

3 × 10^6^ (80% confluency) of the human MM418C1 melanoma cells were lysed using Triton X-100 lysis buffer (1% (*v*/*v*) Triton X-100, 50 mM sodium phosphate buffer, pH 6.8). After that, the mixture was sonicated for 30 s at 30 kHz using an Epishear Sonicator (Carlsbad, CA, USA). The cell lysates were centrifuged for 30 min at 7500× *g* at 4 °C. Protein estimation was used to determine the amount of protein in each cell sample. Triton X-100 lysis buffer was used as a blank for the TYR activity assay. For each sample, 20 μL of the potential TYR inhibitors (1 mg/mL, 5% DMSO was used to assist solubilization of certain extracts and compounds) were added to a 96-well plate in triplicate, followed by 160 μL of 15 mM L-Dopa solution in 0.1 M sodium phosphate buffer (0.049 M Na_2_HPO_4_·7H_2_O, 0.051 M NaH_2_PO_4_·H_2_O). Finally, 20 μL of cell lysate was added to each well. Inhibitors can be added to specific wells at this stage. A multi-mode plate reader (BMG Labtech, Ortenberg, Germany) was used to measure the change in absorbance at 475 nm over 60 min.

TYR activity (% remaining) can be calculated using the following equation:

$$\;TYR\;activity\;\left(\%\right)=\frac{\left(A1-A2\right)}{\left(A3-A4\right)}\;*\;100\%\;$$

L-DOPA solution and sodium phosphate buffer were used as a blank (A1);

L-DOPA and cell lysate containing *hs*TYR without test sample were used as a negative control (A2);

L-DOPA and cell lysate containing *hs*TYR with the test sample were the test reactions (A3);

L-DOPA and the test sample without cell lysate were used as a negative control (A4) [12].

### 4.7. Resazurin Cell Proliferation Assay

Cells were seeded at a density of 30,000 cells per well in a 96-well plate and incubated overnight at 37 °C with 5% CO_2_. The following day, cells were treated with specific drug concentrations, bringing the total volume to 100 μL per well, and treatments were performed in triplicate. Growth media without cells served as a vehicle control (blank), while cells treated with 10% DMSO were used as a positive control. Following treatment, the cells were incubated at 37 °C, 5 μL of CellTiter-Blue^®^ (Resazurin) Reagent (Promega Corp, Sydney, NSW, Australia) was added to each well, and the plate was mixed for 10 s using a plate reader. The cells were subsequently incubated for 3 h at 37 °C in a 5% CO_2_ environment. Fluorescence was then measured at 560 nm excitation and 590 nm emission using the FLUOstar Omega multi-mode plate reader (BMG Labtech, Ortenberg, Germany).

### 4.8. Melanin Assay

Melanin content of human MM418C1 melanoma cells was measured following the previous method with some modifications [57]. Cells (0.5–1 × 10^5^) were seeded in a 6-well plate with 780 μL culturing medium and incubated overnight at 37 °C with 5% CO_2_. The following day, cells were treated with the appropriate concentration of inhibitors and 120 μL Alpha-melanocyte-stimulating hormone (α-MSH, 0.1 μM) for 5 days. After treatment, cells were washed twice with 1 × PBS and collected by trypsinization or scraping into 80 μL of 1 M NaOH containing 10% DMSO. The cell suspensions were then incubated at 60 °C for 1 h to allow for melanin extraction. Melanin (Sigma, Clayton, VIC, Australia) was dissolved in 1 M NH_4_OH to prepare a 1 mg/mL melanin stock solution. This stock solution was then diluted to prepare melanin standard solutions at concentrations of 80 µg/mL, 40 µg/mL, 20 µg/mL, 10 µg/mL, 5 µg/mL, and 2.5 µg/mL using 1 M NaOH. Subsequently, 100 μL of each melanin standard solution was transferred to a 96-well plate in triplicate to generate a standard curve. The cell extracts were diluted 1:2 with 1 M NaOH, and 100 μL of the diluted cell extracts were transferred in triplicate to the appropriate wells of the 96-well plate. The plate was mixed for 30 s using a SpectraMax M3 plate reader (Molecular Devices, San Jose, CA, USA), and absorbance was measured at 405 nm. The melanin content of each sample was determined using the melanin standard curve. Protein estimation (as described in Section 4.5) was employed to measure the total protein concentration in each cell sample. The melanin content was then normalized by calculating the μg of melanin per mg of total protein for each sample, allowing for a standardized comparison of melanin levels across different samples.

### 4.9. Cellular Tyrosinase Assay

Tyrosinase activity was measured following a modified version of the method [58]. Cells (0.5–1 × 10^6^) were seeded in a 6-well plate and incubated overnight at 37 °C with 5% CO_2_. The next day, cells were treated with the appropriate concentration of the inhibitors and 120 μL α-MSH (0.1 μM). After the treatment period (5 days), cells were washed twice with 1× PBS and collected by trypsinization or scraping into 100 μL of ice-cold lysis buffer containing 1% Triton X-100. The cell suspension was then sonicated at 20 kHz for 30 s. Cell lysates were centrifuged at 7500× *g* for 30 min at 4 °C, and the supernatant was collected and stored at −20 °C for further analysis. For the tyrosinase activity assay, 20 μL of 1% Triton X-100 buffer was used as a blank. Then, 20 μL of each cell lysate was added to a 96-well plate in triplicate, followed by the addition of 150 μL of 15 mM L-Dopa solution. Absorbance was measured at 490 nm at 1 min intervals for 60 min using the FLUOstar (Ortenberg, Germany) Omega multi-mode plate reader (BMG Labtech). Tyrosinase activity was quantified by measuring the absorbance at 490  nm/min/mg protein over 60  min, with the change in absorbance values reflecting the conversion of L-Dopa into dopaquinone. Protein concentration for each cell sample was determined by protein estimation (Section 4.5).

### 4.10. Zebrafish Maintenance and Depigmentation Assay

Adult zebrafish and embryos were obtained from ZebraClinic (Institute for Biomedicine and Glycomics, Brisbane, QLD, Australia) maintained by standard protocols approved by Griffith University Animal Ethics Committee. Ethic approval GRIDD/11/22/AEC. Wild-type lines used in these studies are AB background. AB embryos were produced via pair mating, then distributed into 24-well plates at a density of 10 embryos per well at 1 h post-fertilization (hpf). The embryos were supplemented with standard E3 medium (5 mM NaCl, 0.17 mM KCl, 0.33 mM CaCl_2_, 0.33 mM MgSO_4_), then treated with compounds/extracts at 8 hpf. Kojic acid (4 mg/mL) was used as positive control and standard E3 medium was the negative control. All the embryos were incubated for 48 h prior to analysis. From each treatment group, 9 embryos were randomly chosen, collected and anesthetized using 0.016% tricaine in E3 medium to immobilize them for imaging. Following anaesthetization, embryos were carefully transferred into 1–1.5% methylcellulose prepared in E3 medium and mounted onto a glass-bottom dish for live imaging. Pigmentation was both qualitatively and quantitatively assessed using an Olympus MVX10 mounted with a DP74 camera (Olympus, VIC, Australia). Quantifications were conducted using Fiji software. The images were converted to 8-bit grayscale format and then the entire caudal fin was outlined using freehand selection tool. The black pigmentation within the entire caudal fin and the overall area of the entire caudal fin were calculated using the measure and thresholding tools. Finally, the percentage of pigmentation was analyzed using Graphpad Prism 9 software. Embryos treated with 4 mg/mL kojic acid were utilized as the positive control and embryos treated with standard E3 medium were utilized as the negative control.

## 5. Conclusions

In summary, this study identified 4HB from *Xanthium strumarium* L. as a potent *hs*TYR inhibitor with an IC_50_ of 57.14 ± 5.25 μg/mL in human melanoma MM418C1 cell lysate assay, comparable to kojic acid (IC_50_ = 67.07 ± 12.22 μg/mL). In human MM418C1 melanoma cells, 4HB reduced melanin content by 33% at 500 μg/mL without notable cytotoxicity. In zebrafish models, 4HB achieved a 40% pigmentation reduction at 15.63 μg/mL, though toxicity increased at higher concentrations. Other compounds, including thymidine (**3**), caffeoyl choline (**5**), 5-caffeoylquinic acid (**7**) and 1, 3-dicaffeoylquinic acid (**11**), contributed to *hs*TYR inhibition, supporting the potential of *X. strumarium* methanol extract as a depigmenting agent.

## Data Availability

Data are contained within the article and Supplementary Materials.

## Supplementary Materials

The following supporting information can be downloaded at: https://www.mdpi.com/article/10.3390/molecules30183689/s1.
