# Supplementary material for: Isolation and Biological Evaluation of Human Tyrosinase Inhibitors from the Fruit of Xanthium strumarium L
Source: Molecules. 2025 Sep 10;30(18):3689. doi: 10.3390/molecules30183689 (PMC12473028; doi:10.3390/molecules30183689)
Supplement: Supplementary file 1 [file molecules-30-03689-s001.zip › molecules-3836690-supplementary.pdf]

Isolation and Biological Evaluation of Human Tyrosinase Inhibitors from the Fruit of *Xanthium strumarium* L.

Gengxuan Shi [1,2], Yaoying Lu [1], Yougang Zhang [1,2], Ke Zheng [2], Jean Giacomotto [1,2,3], Kathryn F. Tonissen [1,2] and Yunjiang Feng [1,2,*]

[1] Institute for Biomedicine and Glycomics, Griffith University, Nathan, QLD 4111, Australia; maxxie.shi@griffithuni.edu.au (G.S.); y.lu@griffith.edu.au (Y.L.); yougang.zhang@griffithuni.edu.au (Y.Z.); j.giacomotto@griffith.edu.au (J.G.); k.tonissen@griffith.edu.au (K.F.T.)

[2] School of Environment and Science, Griffith University, Nathan, QLD 4111, Australia; ke.zheng@griffithuni.edu.au

[3] Queensland Brain Institute, The University of Queensland, Brisbane, QLD 4067, Australia

* Correspondence: y.feng@griffith.edu.au

List of Figures

Zebrafish pigmentation assay: 4

Figure S1. survival rate of zebrafish with treatment of 4-hydroxybenzoic acid (A) and kojic acid (B).. 4

Figure S2. Timeframe of the zebrafish pigmentation assay and timepoint for each analysis/quantification. 4

Chemistry data: 5

Figure S3. LCMS data for 4-hydroxybenzoic acid (1) 5

Figure S4. LCMS data for uridine (2).. 6

Figure S5. LCMS data for thymidine (3).. 7

Figure S6. LCMS data for cytidine (4) 8

Figure S7. LCMS data for caffeoyl choline (5) 9

Figure S8. LCMS data for 3-caffeoylquinic acid (6).. 10

Figure S9. LCMS data for 5-caffeoylquinic acid (7).. 11

Figure S10. LCMS data for indole-3-carboxaldehyde (8).. 12

Figure S11. LCMS data for xanthiside (9).. 13

Figure S12. LCMS data for 1, 5-dicaffeoylquinic acid (10).. 14

Figure S13. LCMS data for 1, 3-dicaffeoylquinic acid (11).. 15

Figure S14. ^{1}H NMR spectra (800MHz, DMSO -*d6*) for 4-hydroxybenzoic acid (1)..... 16

Figure S15. ^{1}H NMR spectra (800MHz, DMSO -*d6*) for uridine (2) 16

Figure S16. ^{1}H NMR spectra (800MHz, DMSO -*d6*) for thymidine (3).. 17

Figure S17. ^{1}H NMR spectra (800MHz, DMSO -*d6*) for cytidine (4).. 17

Figure S18. ^{1}H NMR spectra (800MHz, DMSO -*d6*) for caffeoyl choline (5).. 18

Figure S19. ^{1}H NMR spectra (800MHz, DMSO -*d6*) for 3-caffeoylquinic acid (6)........ 18

Figure S20. ^{1}H NMR spectra (800MHz, CD_3OD -*d4*) for 5-caffeoylquinic acid (7)....... 19

Figure S21. ^{1}H NMR spectra (800MHz, DMSO -*d6*) for indole-3-carboxaldehyde (8).. 19

Figure S22. ^{1}H NMR spectra (800MHz, CD_3OD -*d4*) for xanthiside (9) 20

Figure S23. ^{1}H NMR spectra (800MHz, CD_3OD -*d4*) for 1, 5-dicaffeoylquinic acid (10) 20

Figure S24. ^{1}H NMR spectra (800MHz, CD_3OD -d_4) for 1, 3-dicaffeoylquinic acid (11) 21

Zebrafish pigmentation assay:

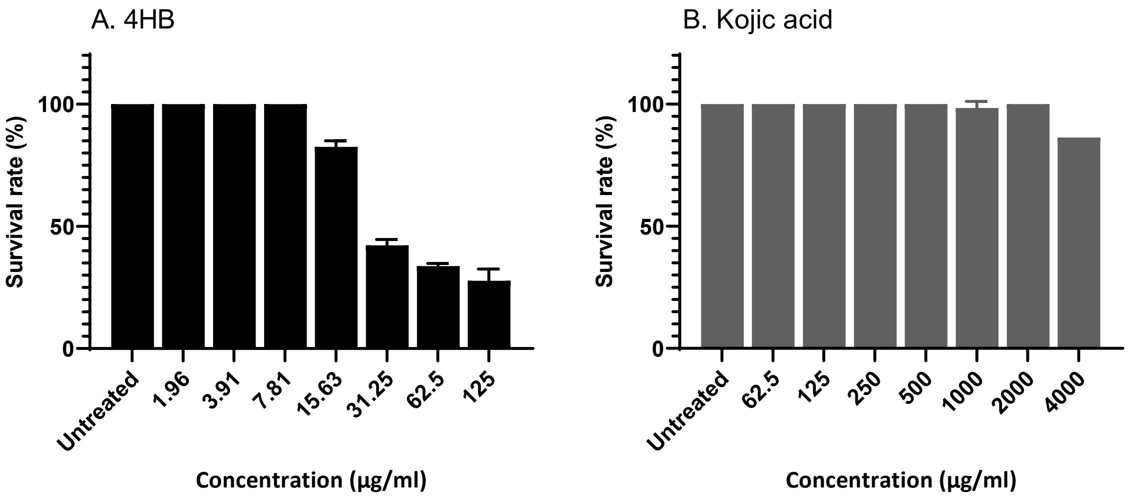

Figure S1. Survival rate of zebrafish with treatment of 4-hydroxybenzoic acid (4HB) (A) and kojic acid (B). 6 hpf zebrafish embryos (n=10) were treated with 125, 62.5, 31.25, 15.63, 7.81, 3.91, 1.96 µg/mL 4HB solution (A) or 4000, 2000, 1000, 500, 250, 125, 62.5 µg/mL kojic acid (B) in E3 media for 48 h. Standard E3 medium was used as the untreated group. Mean ±SEM, each group performed in triplicate are shown.

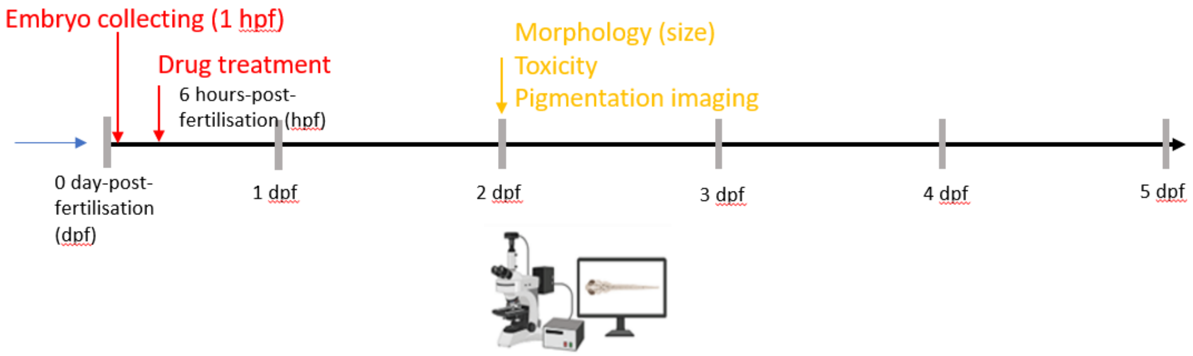

Figure S2. Timeframe of the zebrafish pigmentation assay and timepoint for each analysis/quantification.

Chemistry data:

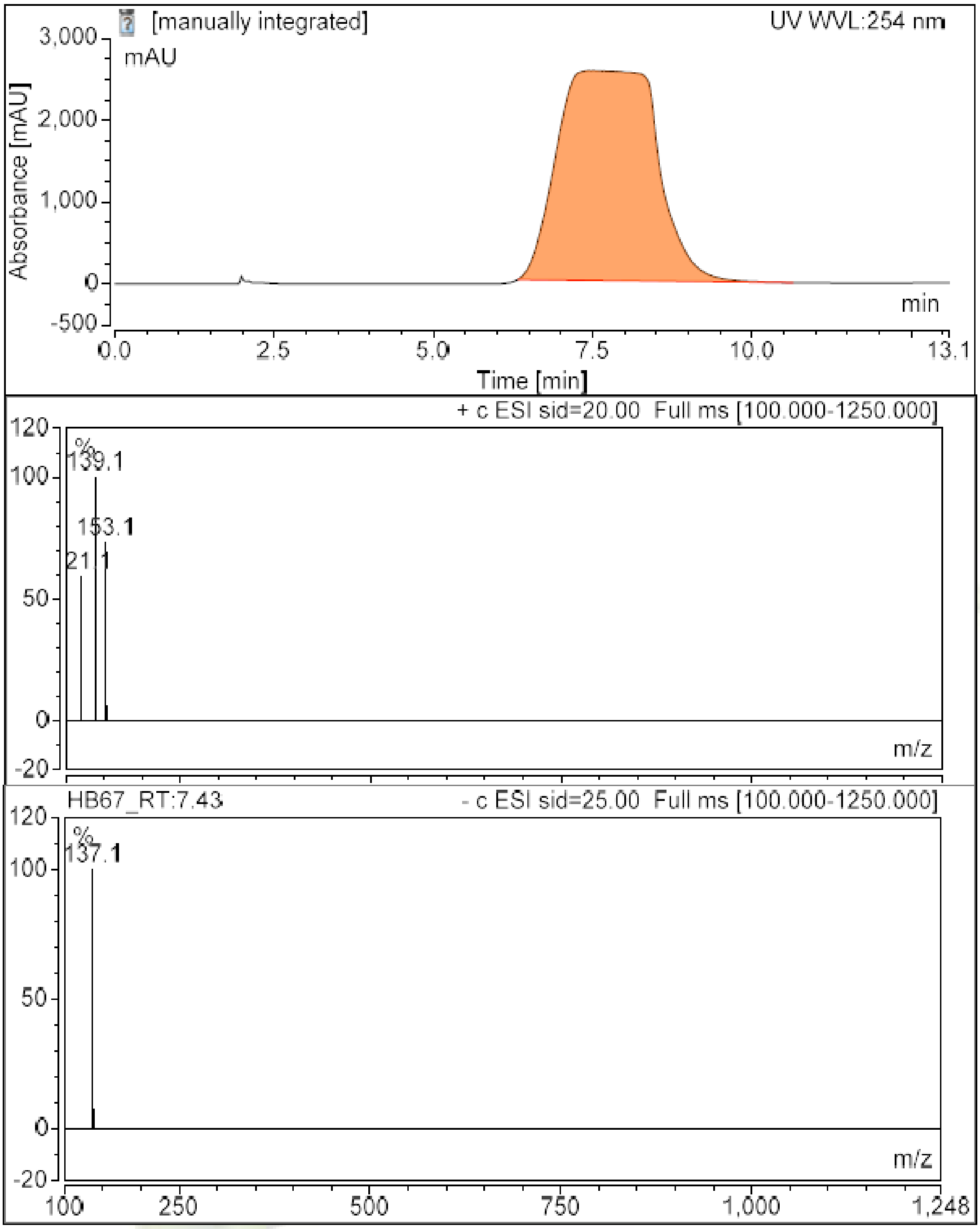

Figure S3. LCMS data for 4-hydroxybenzoic acid (1)

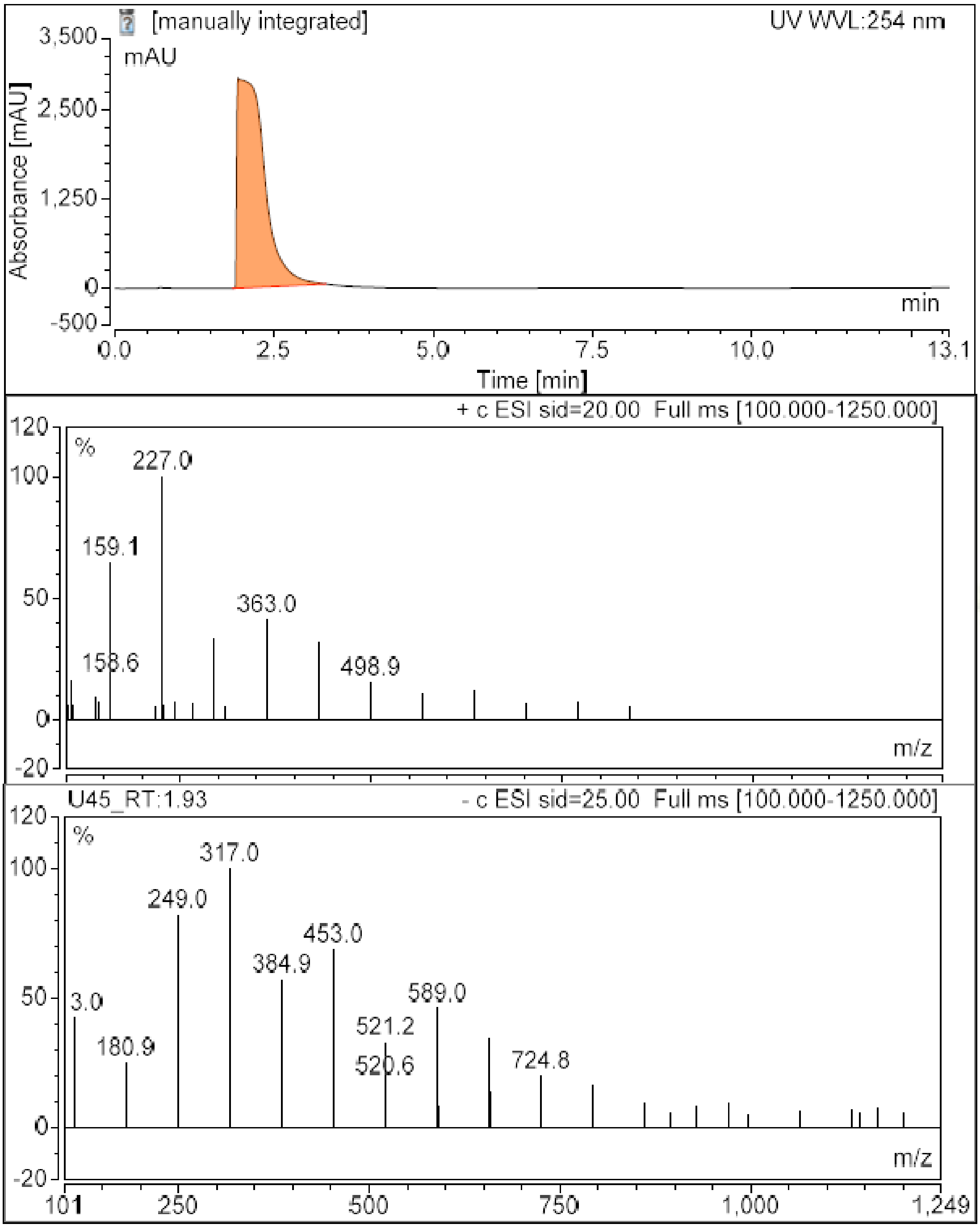

Figure S4. LCMS data for uridine (2)

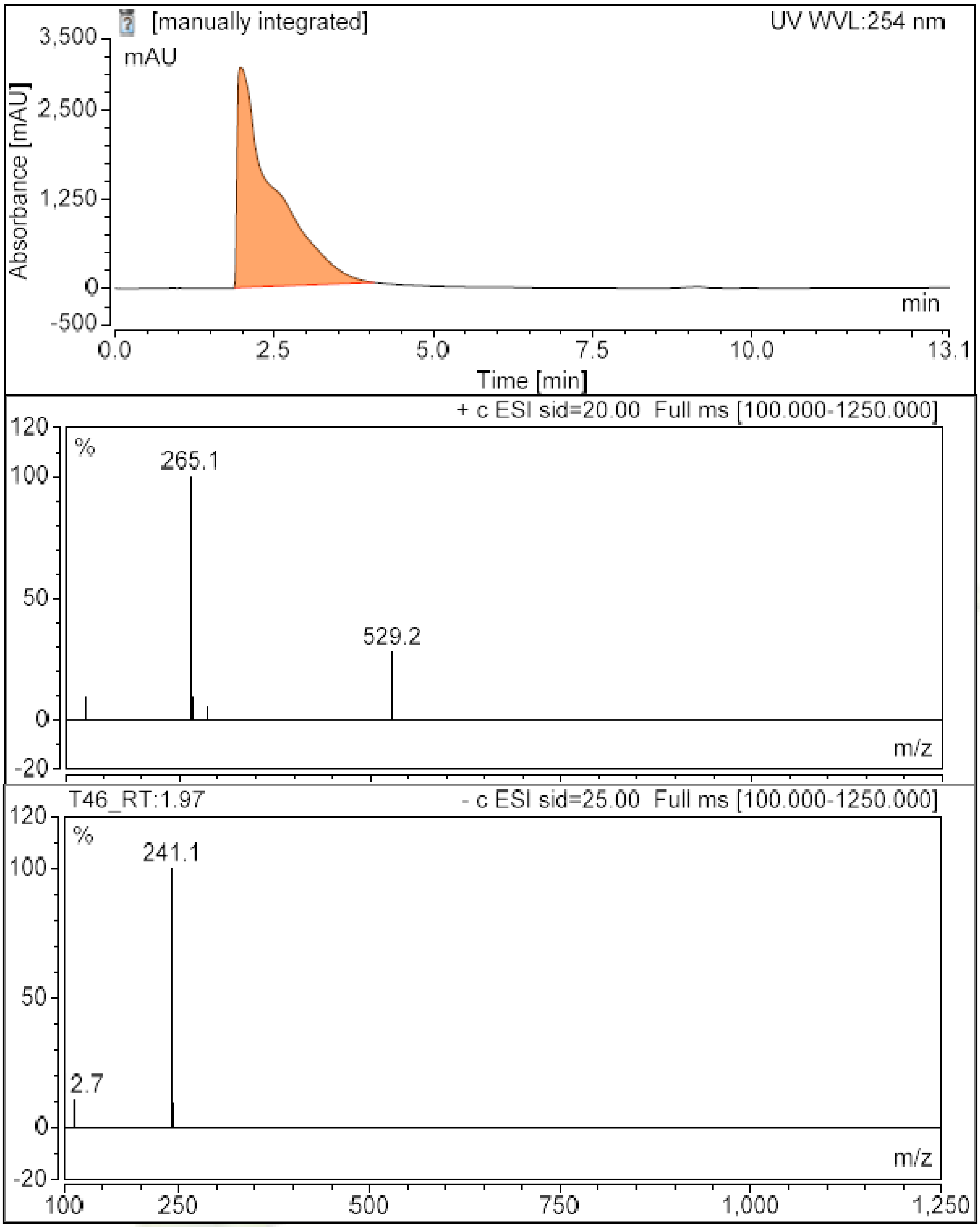

Figure S5. LCMS data for thymidine (3)

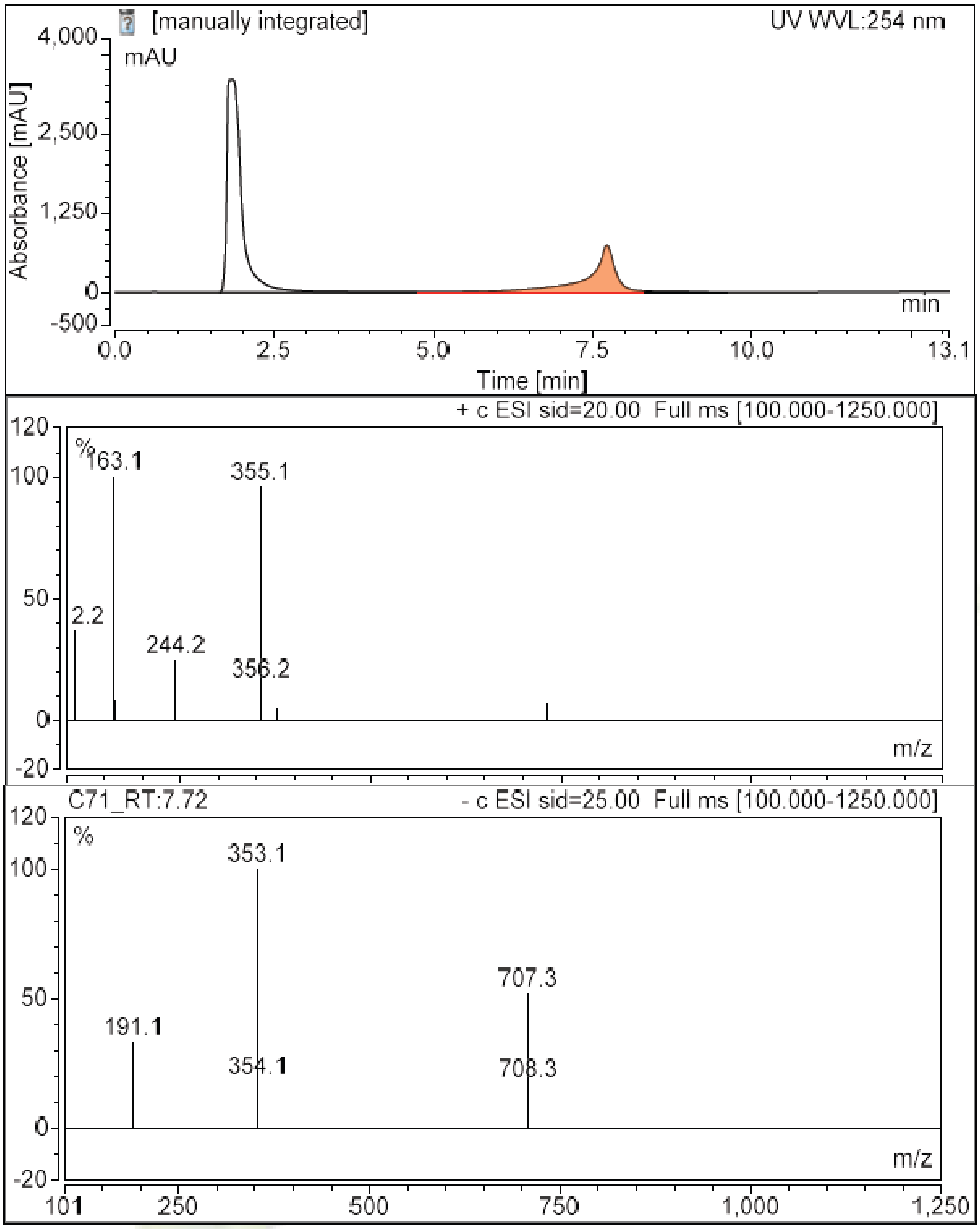

Figure S6. LCMS data for cytidine (4)

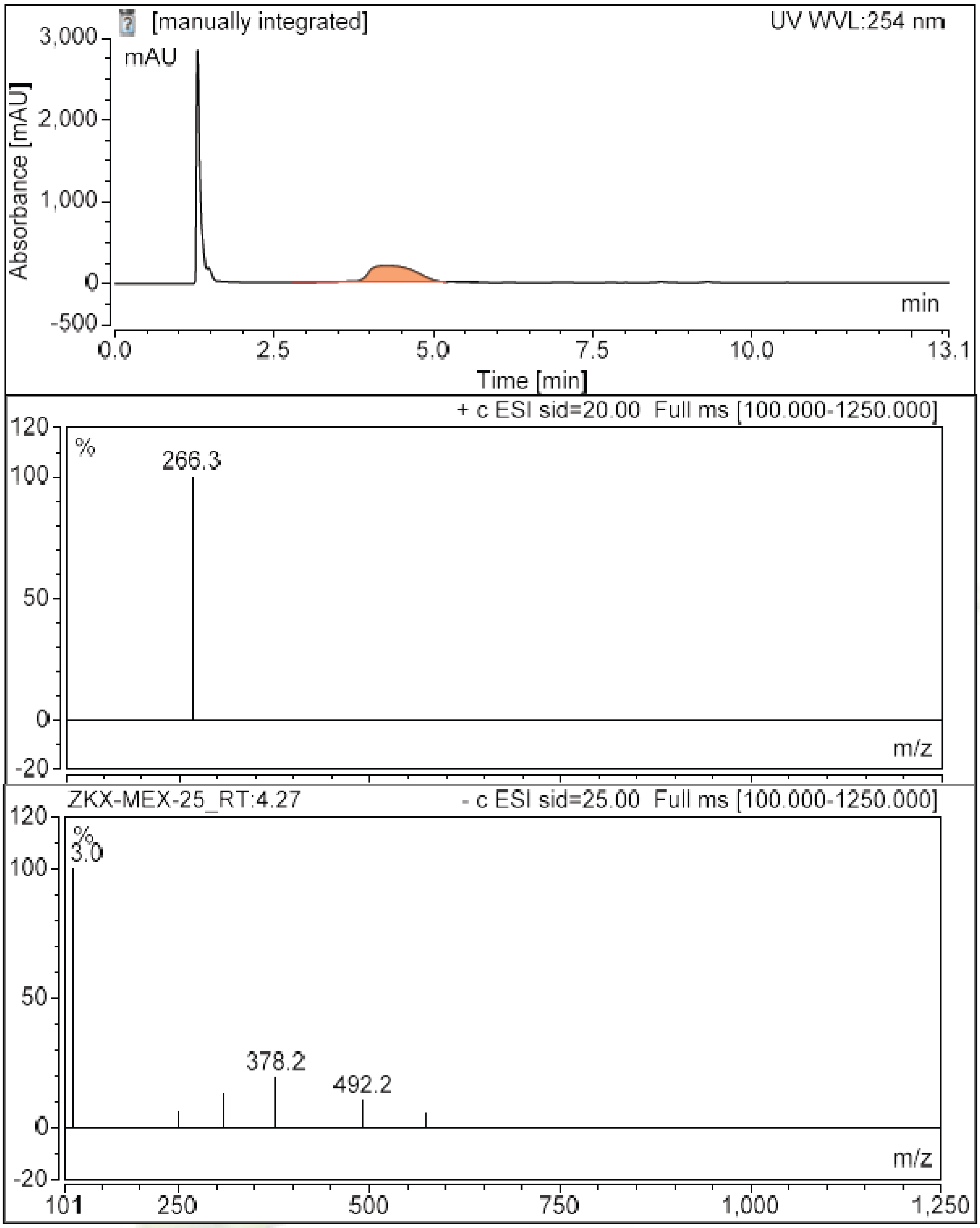

Figure S7. LCMS data for caffeoyl choline (5)

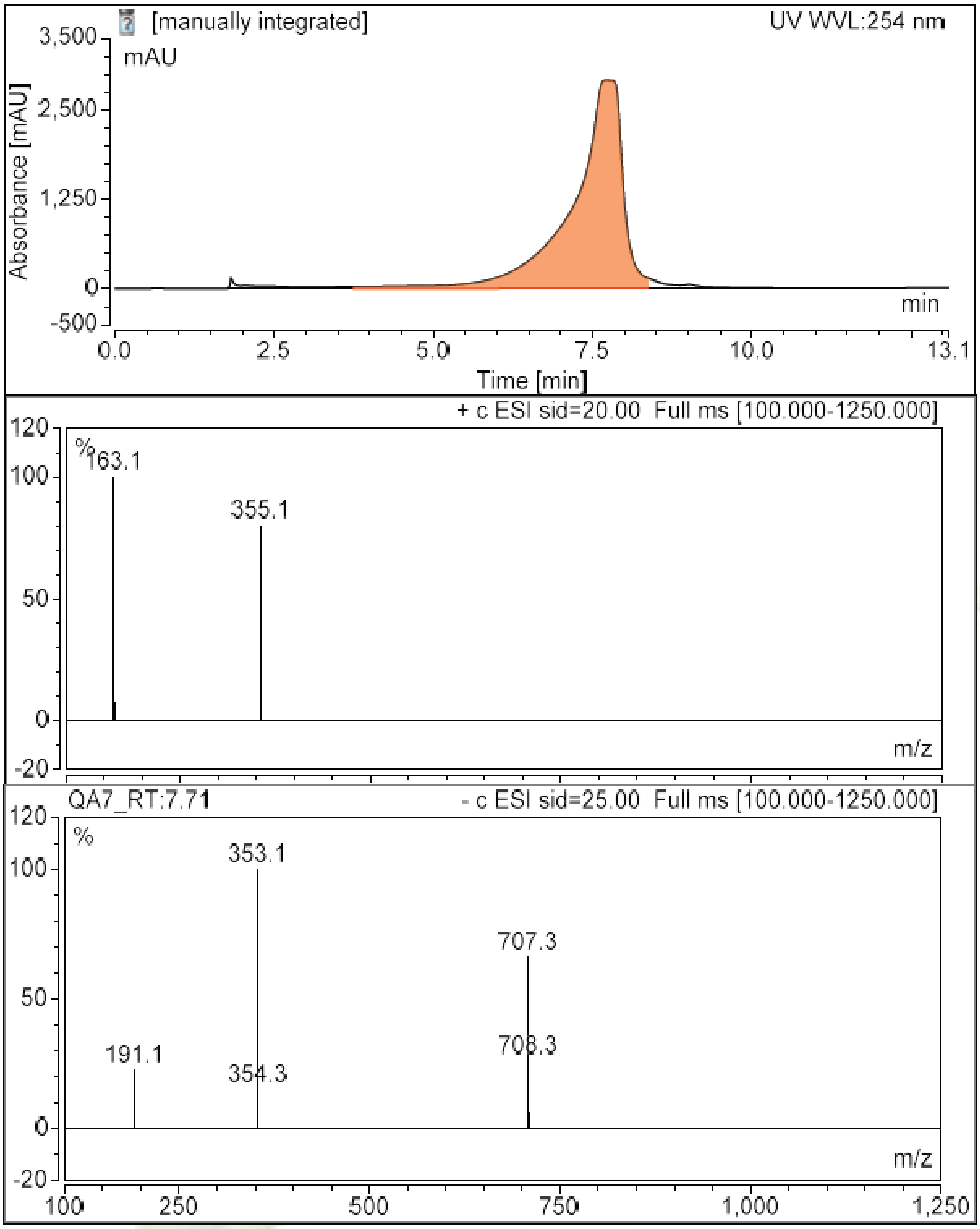

Figure S8. LCMS data for 3-caffeoylquinic acid (6)

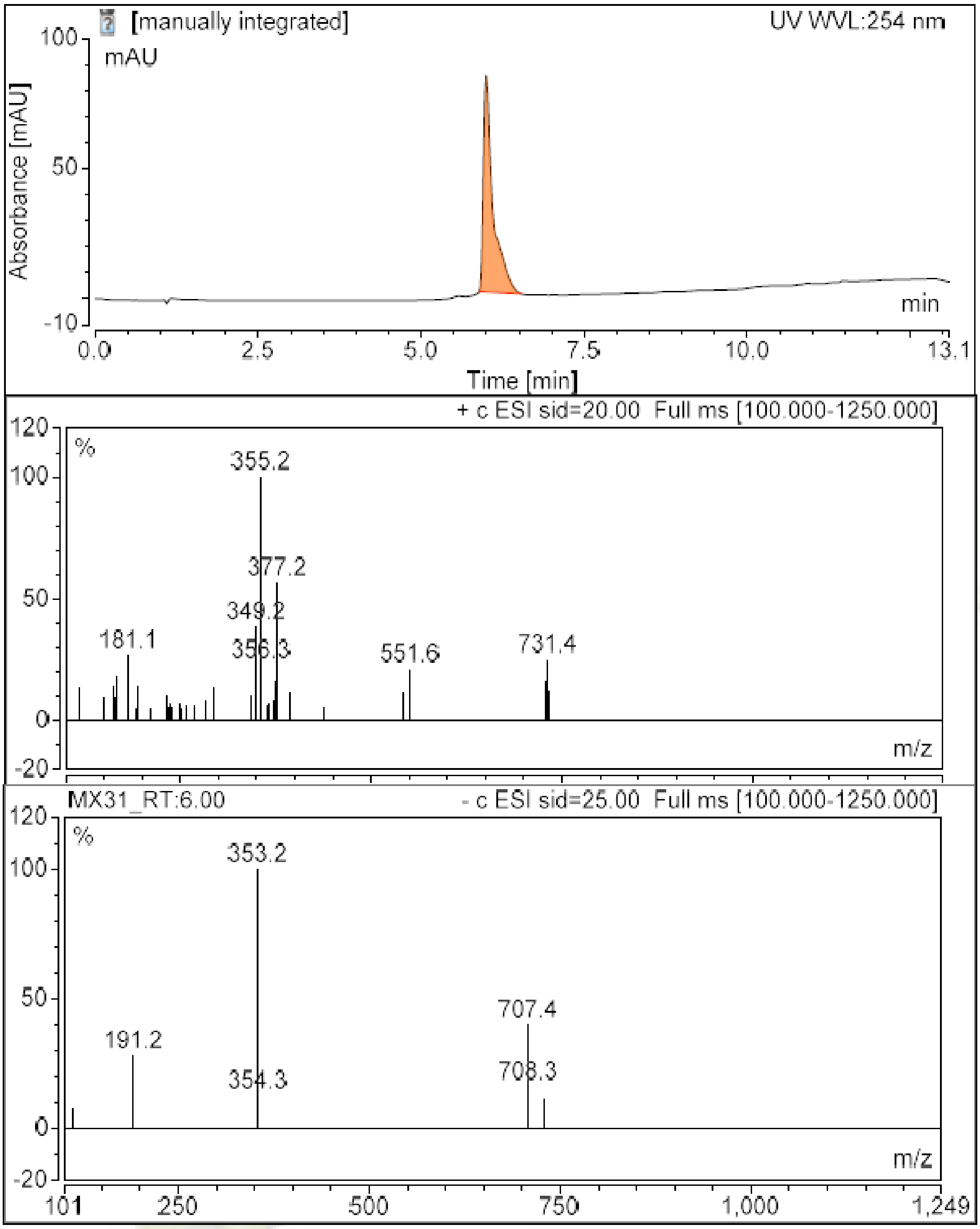

Figure S9. LCMS data for 5-caffeoylquinic acid (7)

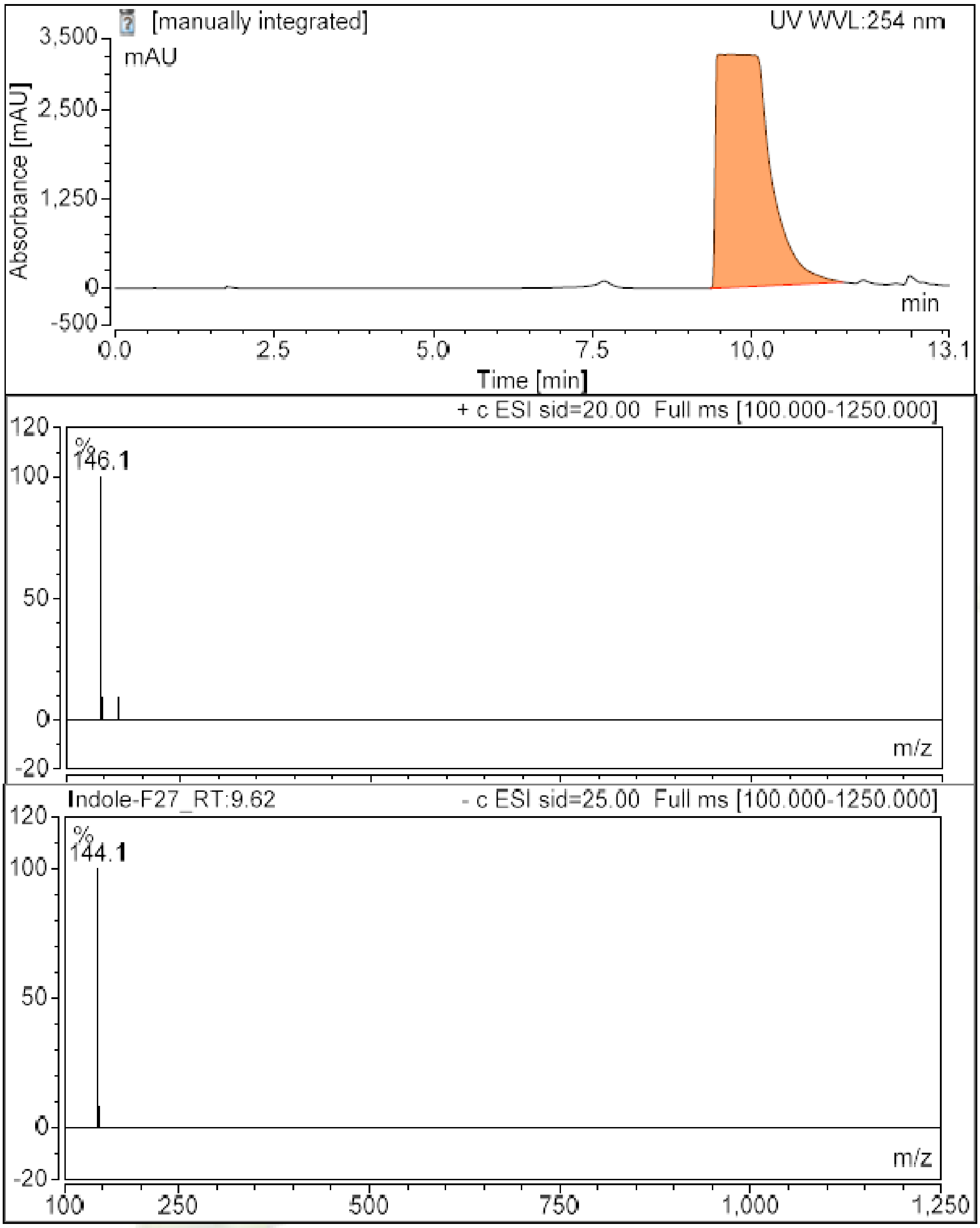

Figure S10. LCMS data for indole-3-carboxaldehyde (8)

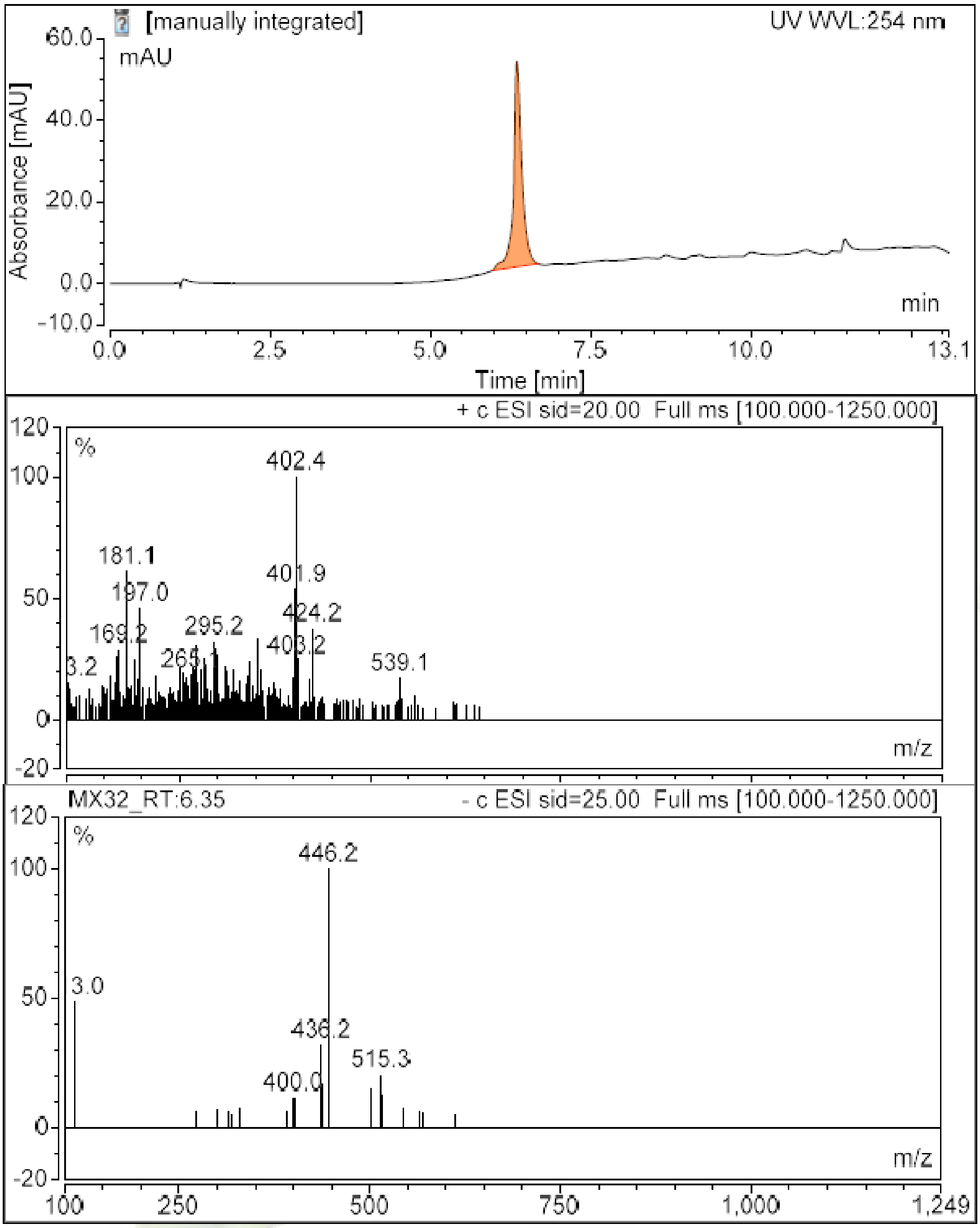

Figure S11. LCMS data for xanthiside (9)

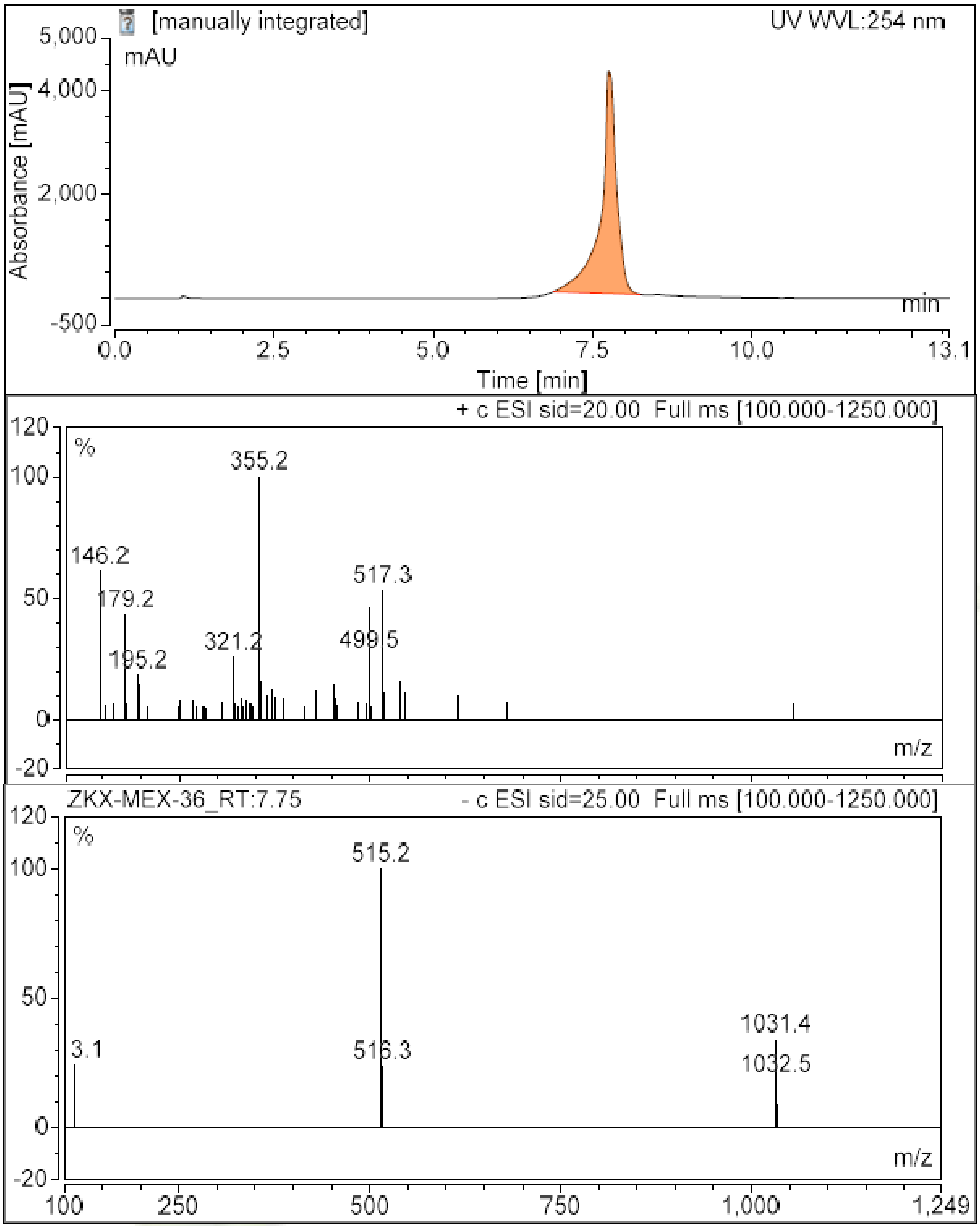

Figure S12. LCMS data for 1, 5-dicaffeoylquinic acid (10)

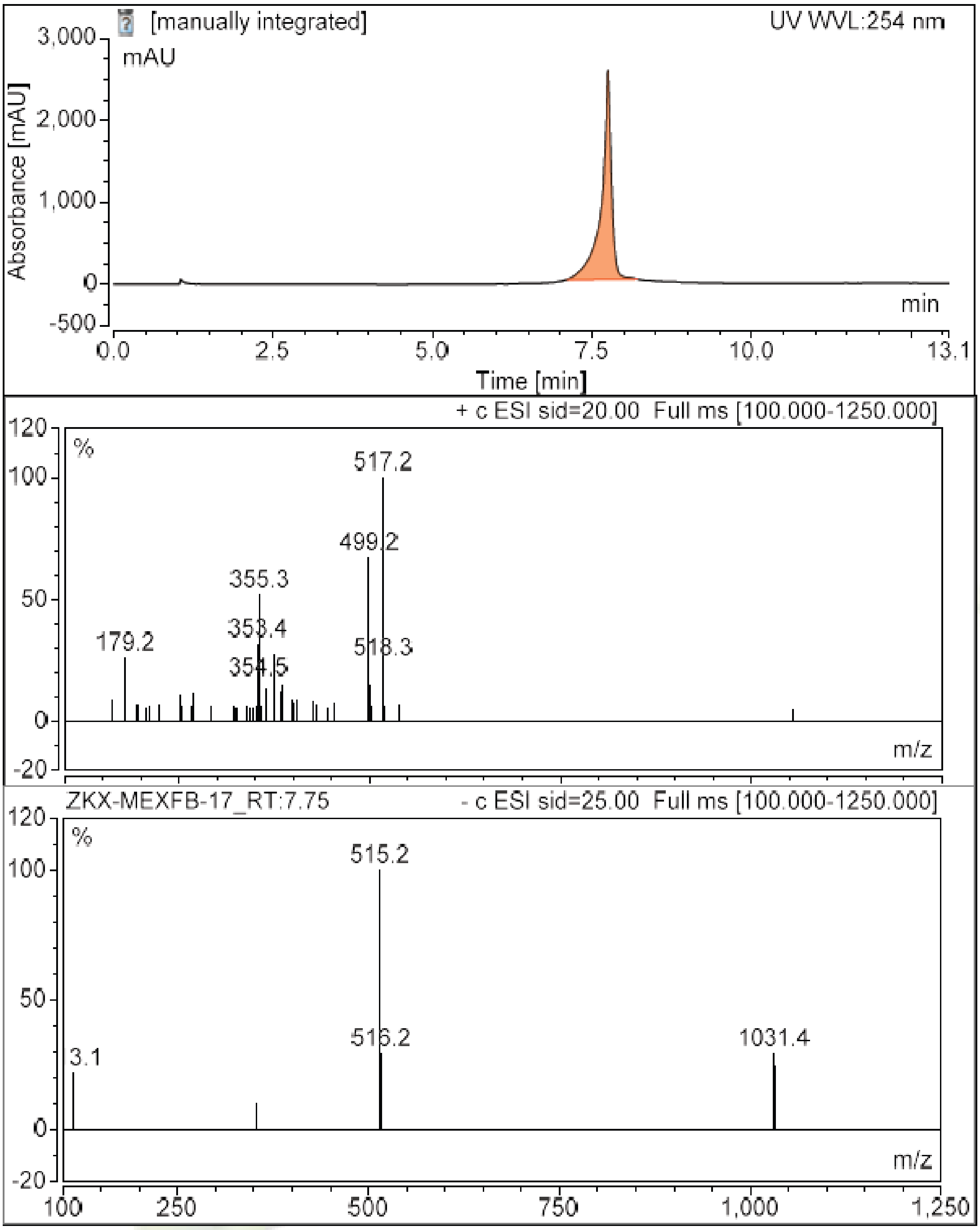

Figure S13. LCMS data for 1, 3-dicaffeoylquinic acid (11)

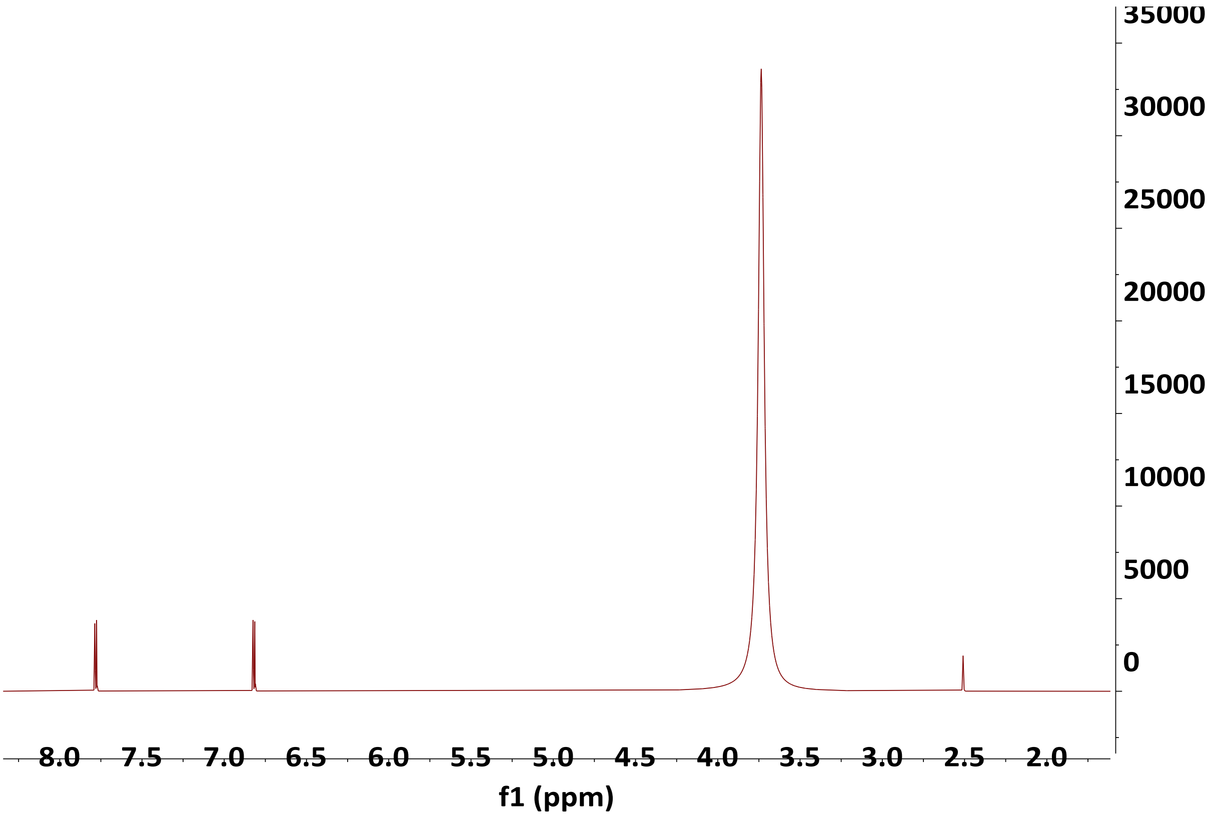

Figure S14. ^{1}H NMR spectra (800MHz, DMSO -*d6*) for 4-hydroxybenzoic acid (1)

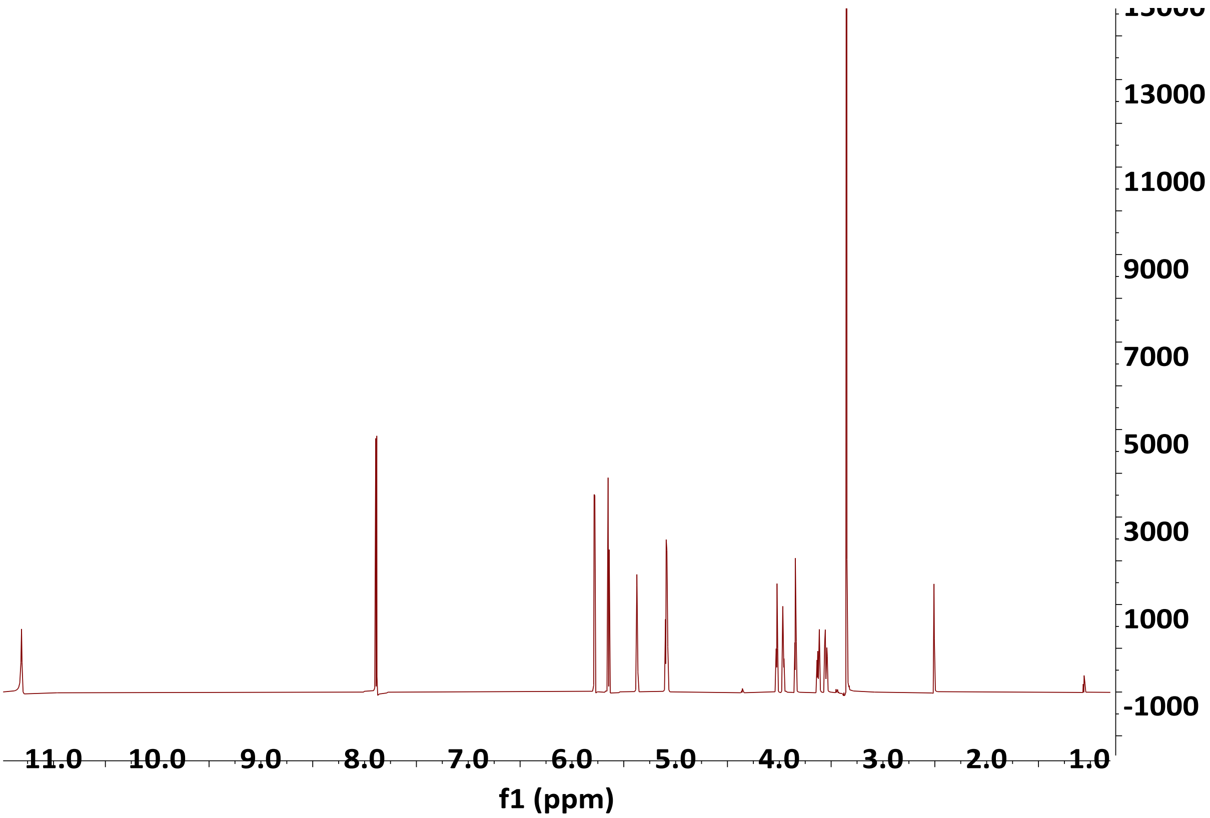

Figure S15. ^{1}H NMR spectra (800MHz, DMSO -*d6*) for uridine (2)

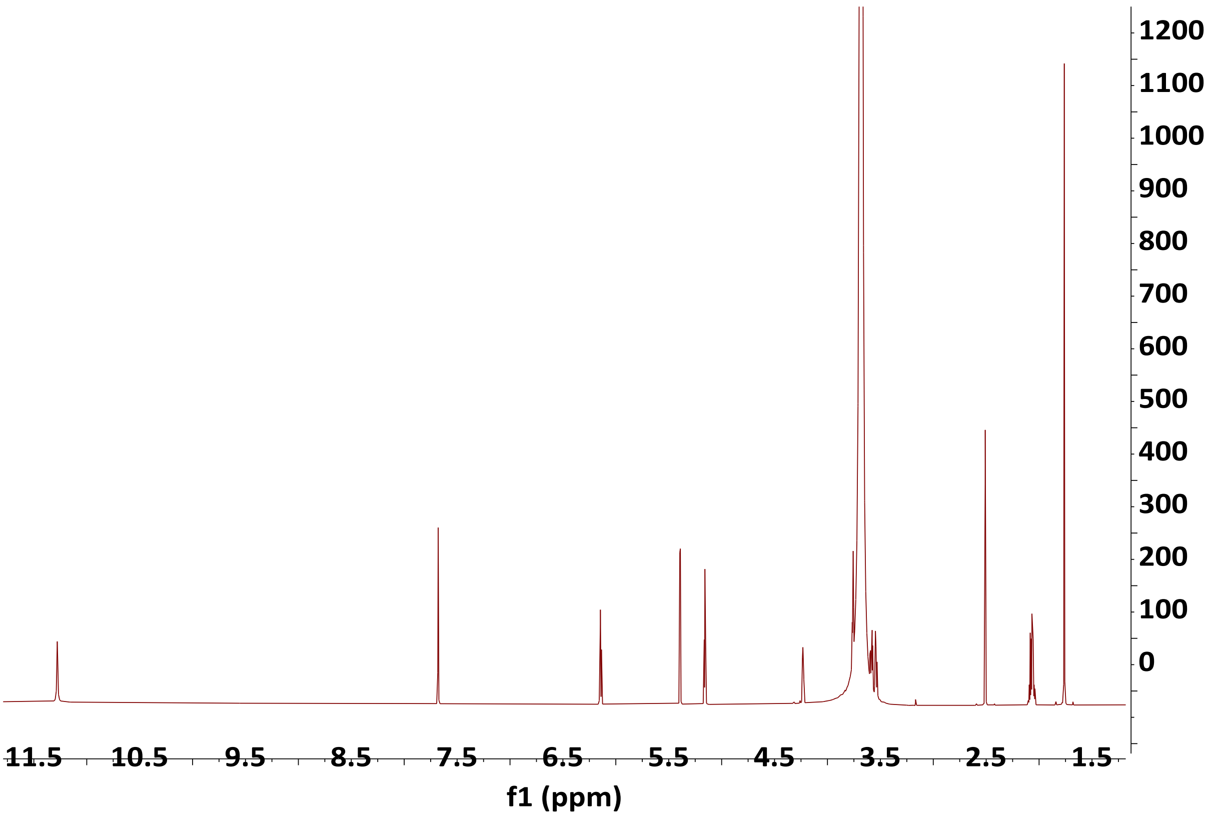

Figure S16. ^{1}H NMR spectra (800MHz, DMSO -d_6) for thymidine (3)

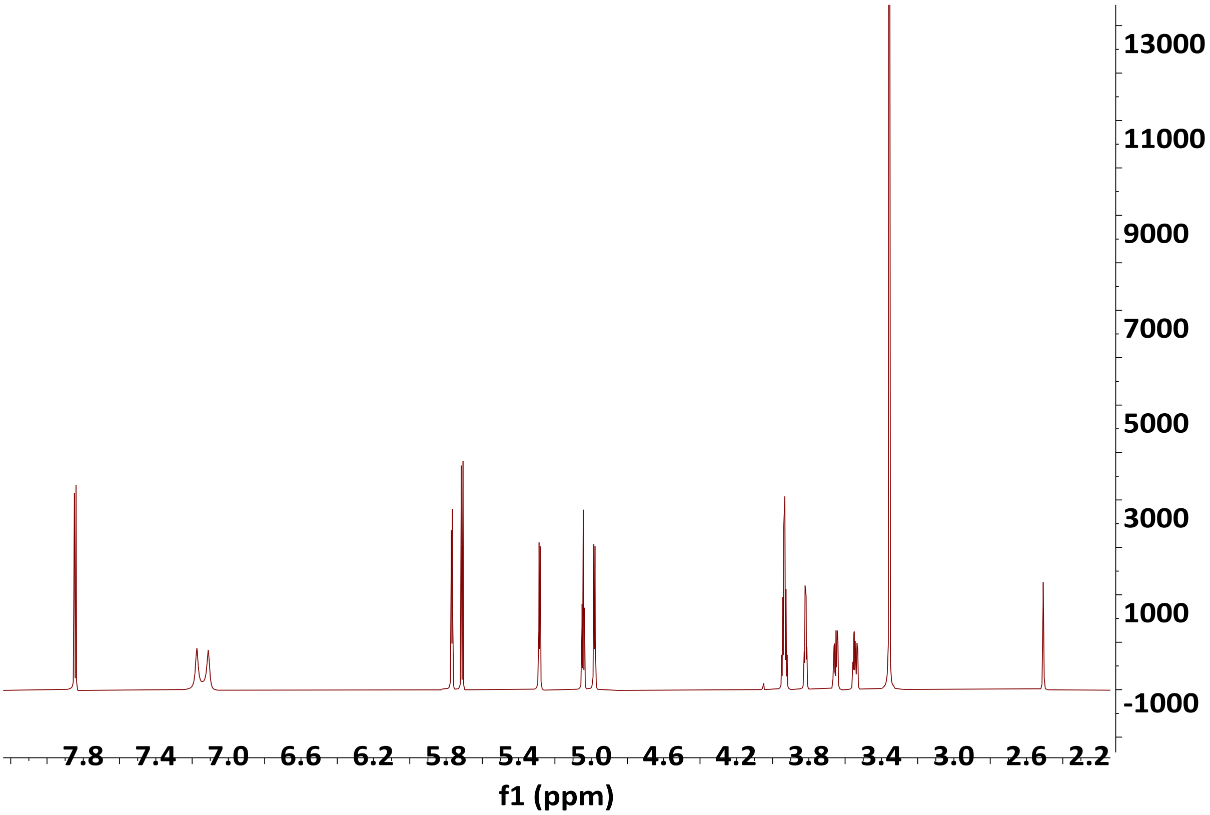

Figure S17. ^{1}H NMR spectra (800MHz, DMSO -d_6) for cytidine (4)

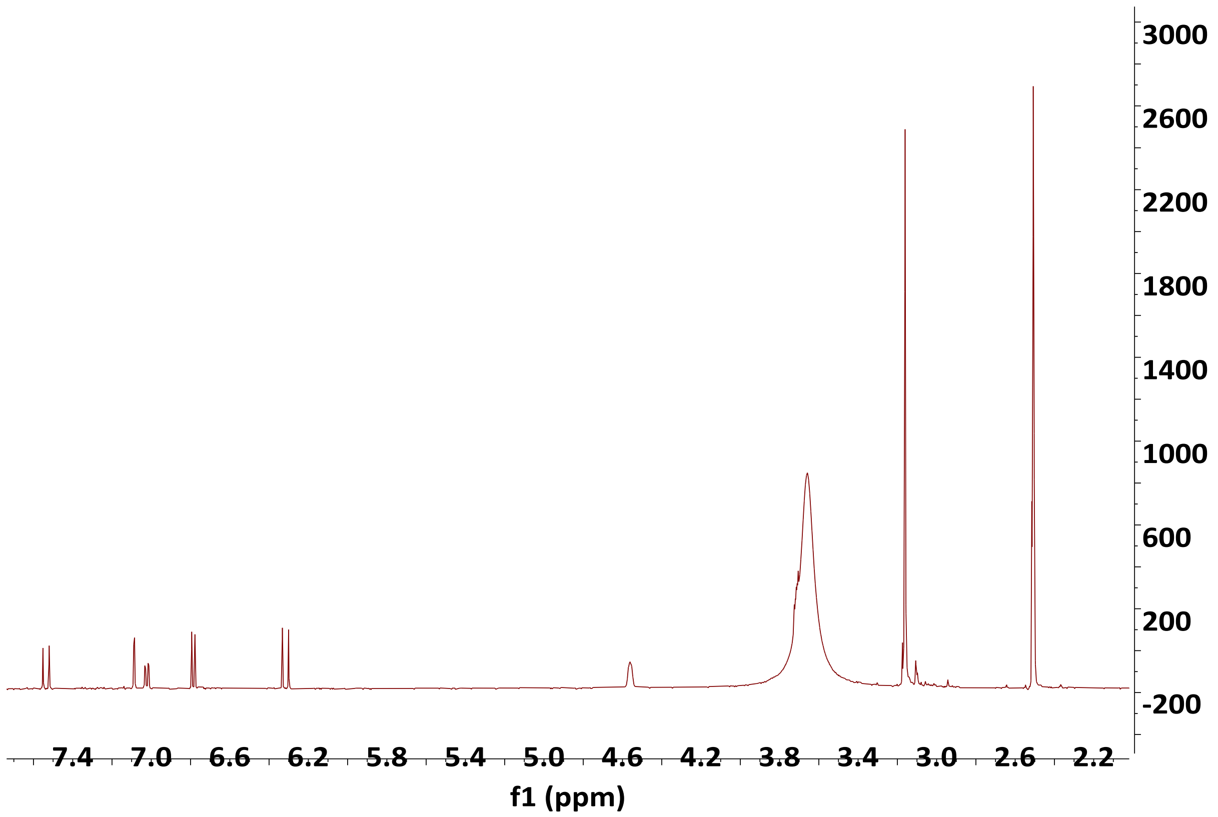

Figure S18. ^{1}H NMR spectra (800MHz, DMSO -*d6*) for caffeoyl choline (5)

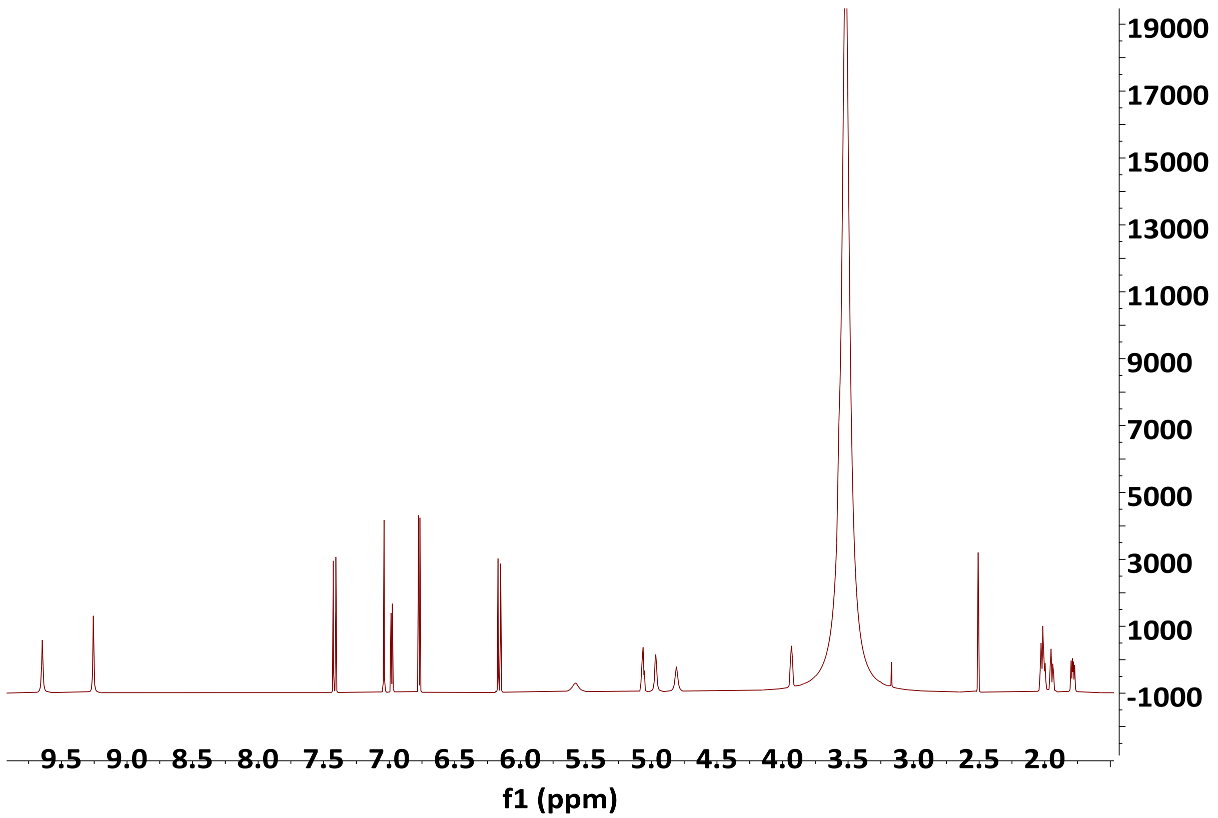

Figure S19. ^{1}H NMR spectra (800MHz, DMSO -*d6*) for 3-caffeoylquinic acid (6)

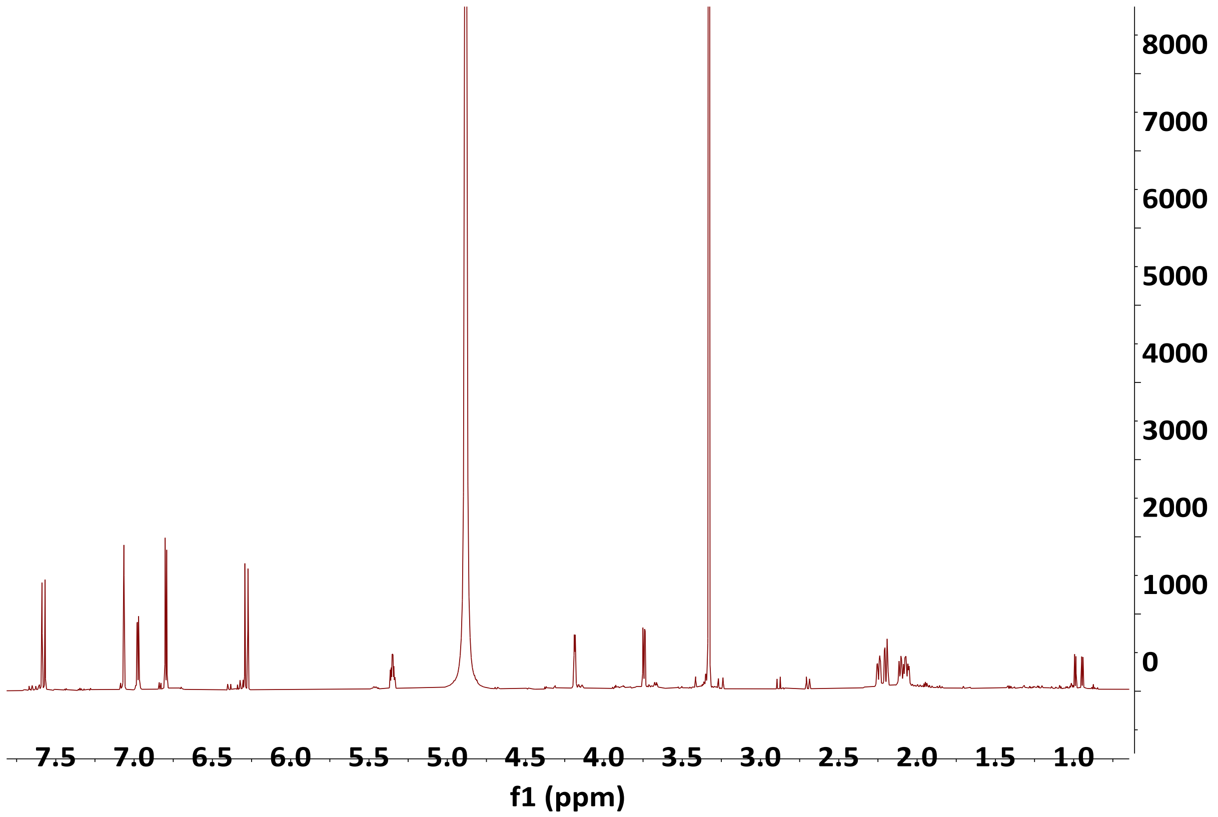

Figure S20. ^{1}H NMR spectra (800MHz, CD_3OD -d_4) for 5-caffeoylquinic acid (7)

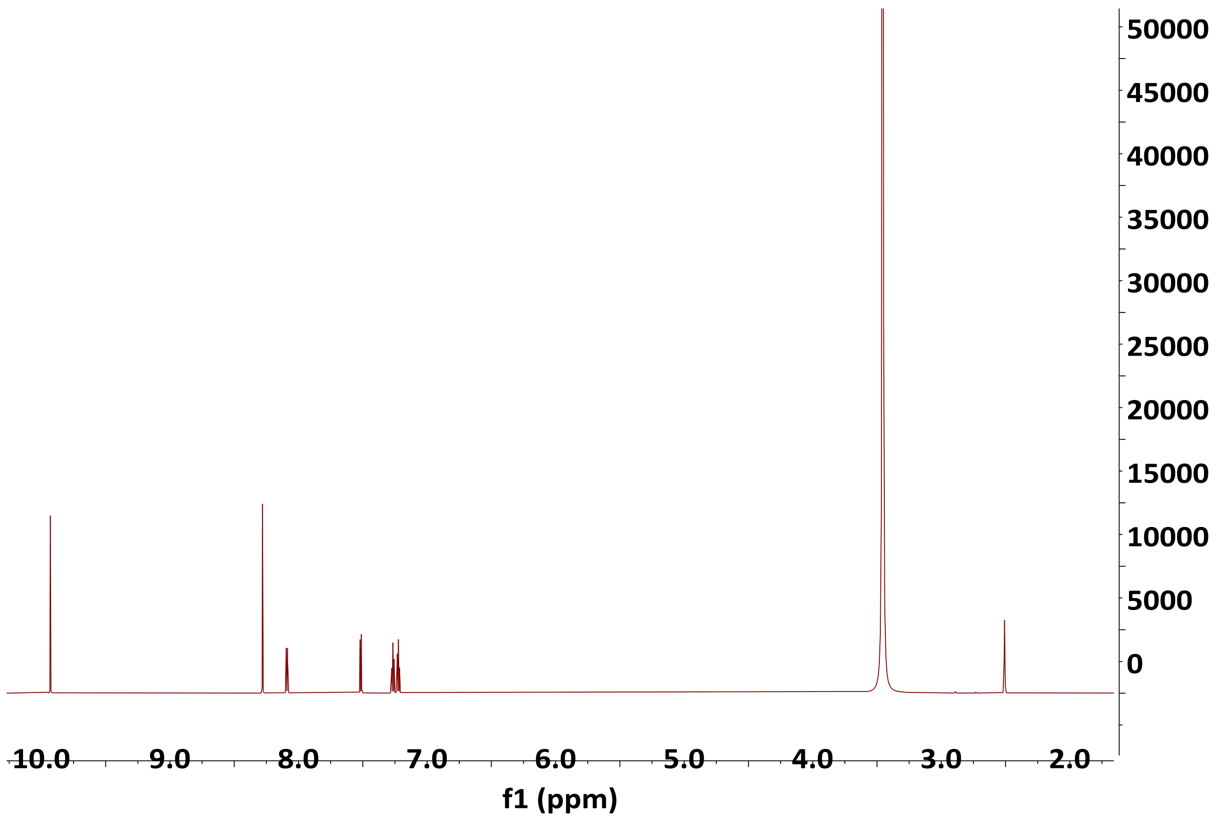

Figure S21. ^{1}H NMR spectra (800MHz, DMSO -d_6) for indole-3-carboxaldehyde (8)

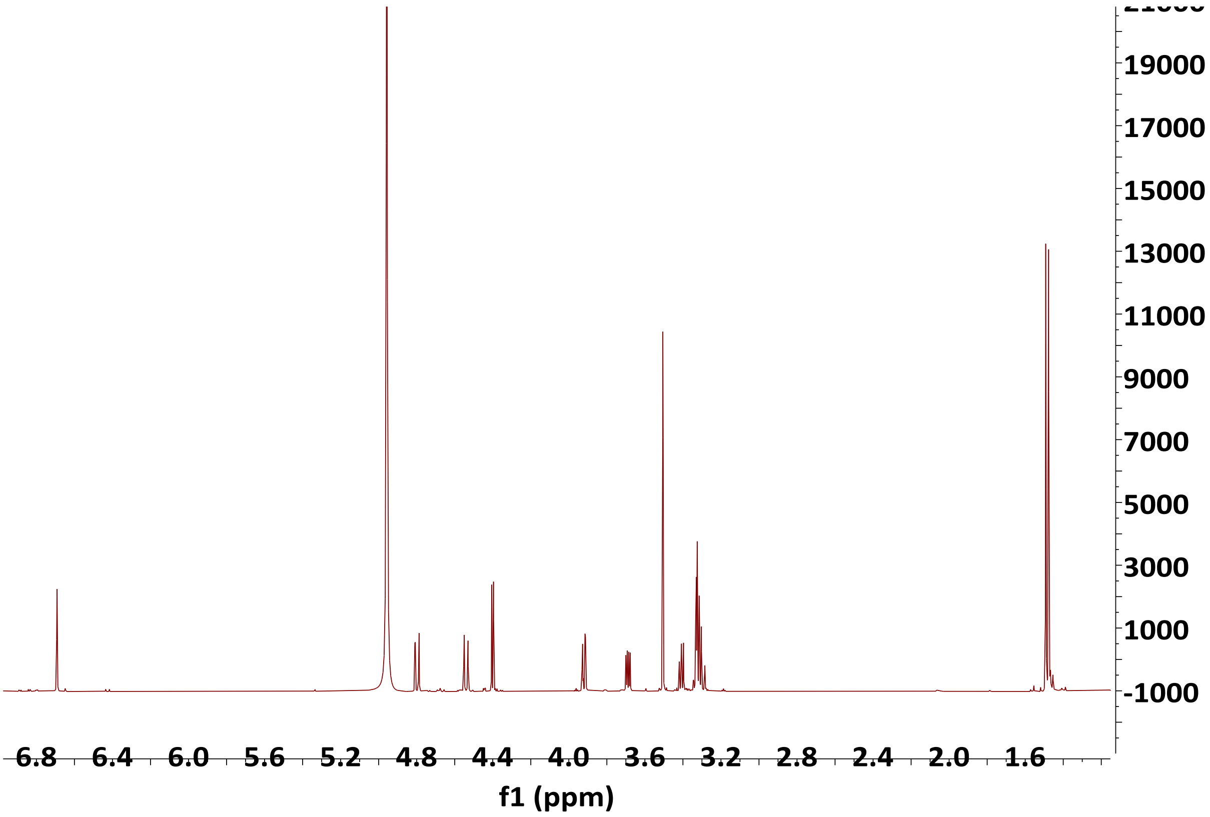

Figure S22. ^{1}H NMR spectra (800MHz, CD_3OD -d_4) for xanthiside (9)

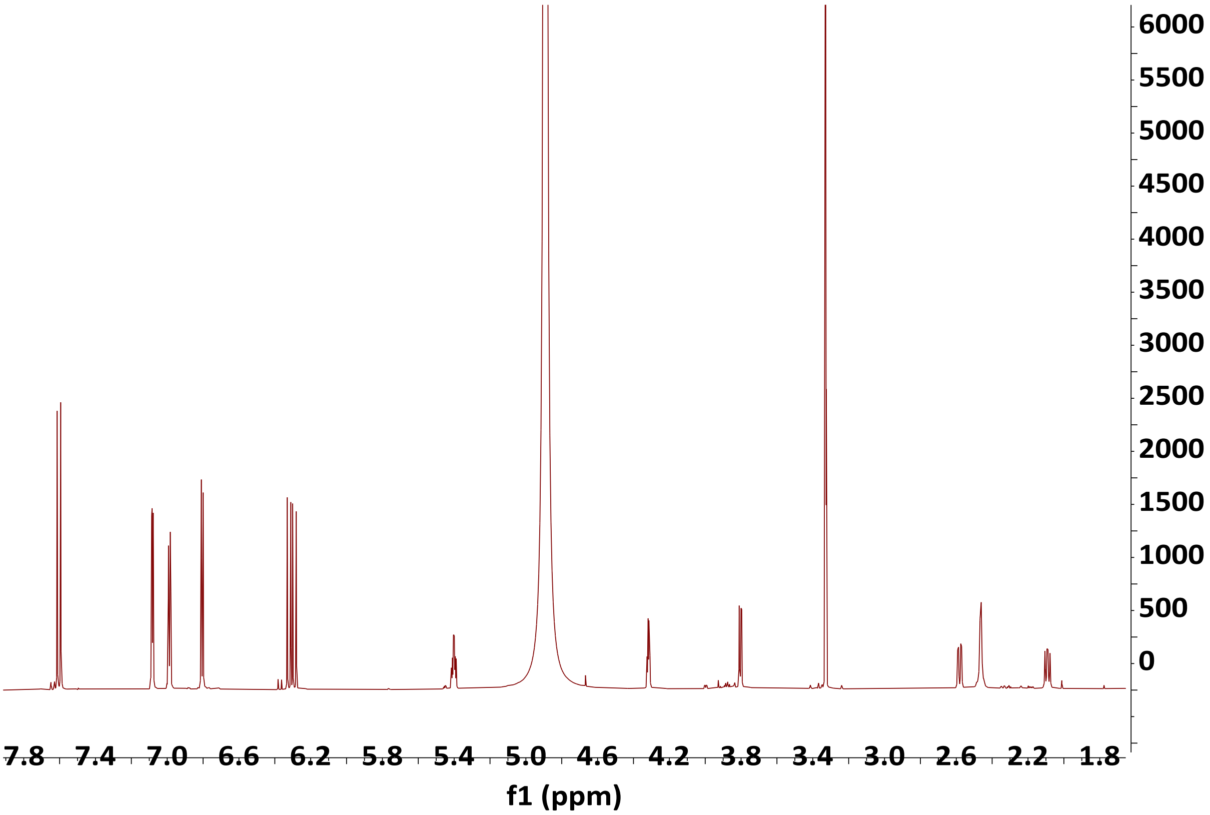

Figure S23. ^{1}H NMR spectra (800MHz, CD_3OD -d_4) for 1, 5-dicaffeoylquinic acid (10)

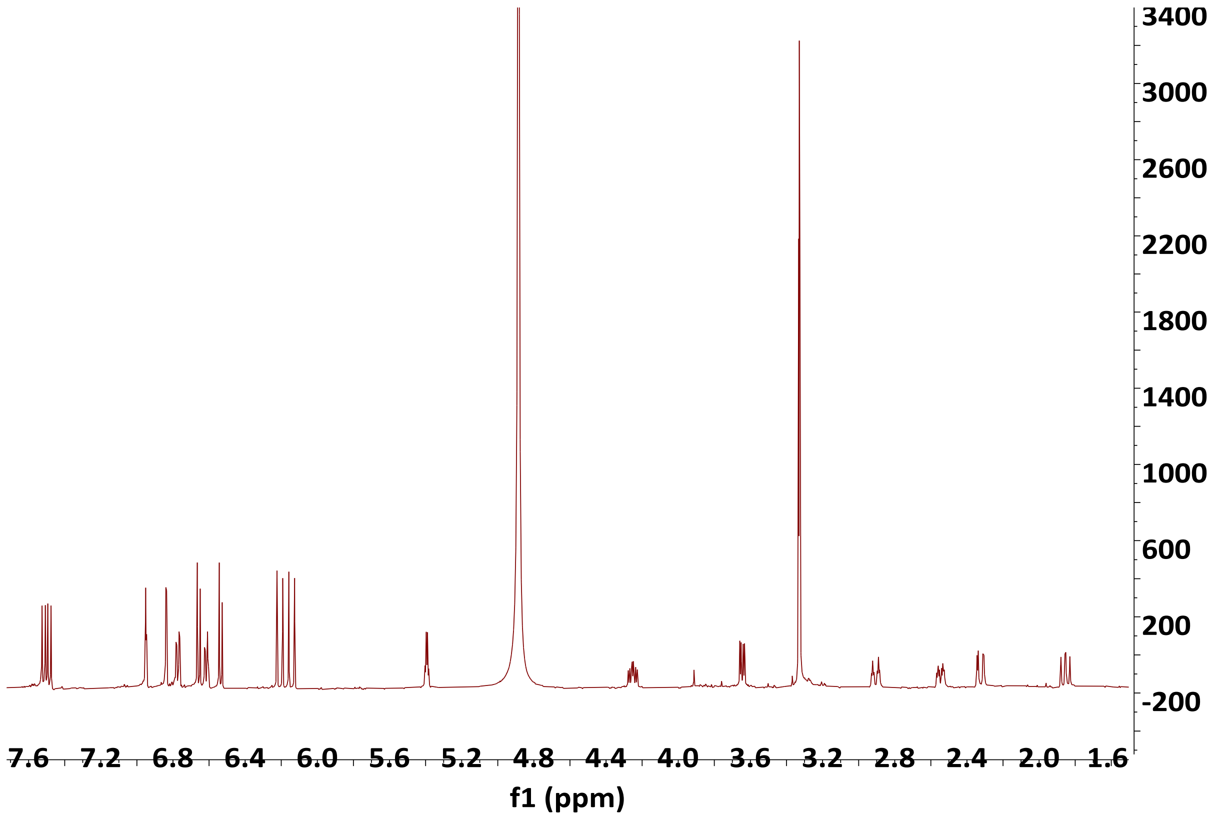

Figure S24. ^{1}H NMR spectra (800MHz, CD_3OD -d_4) for 1, 3-dicaffeoylquinic acid (11)
